# Comparing Digital Versus Face-to-Face Delivery of Systemic Psychotherapy Interventions: Systematic Review and Meta-Analysis of Randomized Controlled Trials

**DOI:** 10.2196/46441

**Published:** 2025-02-24

**Authors:** Pieter Erasmus, Moritz Borrmann, Jule Becker, Lars Kuchinke, Gunther Meinlschmidt

**Affiliations:** 1 Department of Clinical Psychology and Cognitive Behavioral Therapy International Psychoanalytic University Berlin Berlin Germany; 2 Day Clinic for Cognitive Neurology University Hospital Leipzig Leipzig Germany; 3 Psychological Methods and Evaluation International Psychoanalytic University Berlin Berlin Germany; 4 Department of Clinical Psychology and Psychotherapy – Methods and Approaches Trier University Trier Germany; 5 Department of Digital and Blended Psychosomatics and Psychotherapy Psychosomatic Medicine University Hospital and University of Basel Basel Switzerland; 6 Department of Psychology University of Basel Basel Switzerland

**Keywords:** systemic psychotherapy, family therapy, adolescent, systematic review, meta-analysis, face to face, digital, remote, distance, telehealth, delivery modality

## Abstract

**Background:**

As digital mental health delivery becomes increasingly prominent, a solid evidence base regarding its efficacy is needed.

**Objective:**

This study aims to synthesize evidence on the comparative efficacy of systemic psychotherapy interventions provided via digital versus face-to-face delivery modalities.

**Methods:**

We followed PRISMA (Preferred Reporting Items for Systematic Reviews and Meta-Analyses) guidelines for searching PubMed, Embase, Cochrane CENTRAL, CINAHL, PsycINFO, and PSYNDEX and conducting a systematic review and meta-analysis. We included randomized controlled trials comparing mental, behavioral, and somatic outcomes of systemic psychotherapy interventions using self- and therapist-guided digital versus face-to-face delivery modalities. The risk of bias was assessed with the revised Cochrane Risk of Bias tool for randomized trials. Where appropriate, we calculated standardized mean differences and risk ratios. We calculated separate mean differences for nonaggregated analysis.

**Results:**

We screened 3633 references and included 12 articles reporting on 4 trials (N=754). Participants were youths with poor diabetic control, traumatic brain injuries, increased risk behavior likelihood, and parents of youths with anorexia nervosa. A total of 56 outcomes were identified. Two trials provided digital intervention delivery via videoconferencing: one via an interactive graphic interface and one via a web-based program. In total, 23% (14/60) of risk of bias judgments were *high risk*, 42% (25/60) were *some concerns*, and 35% (21/60) were *low risk*. Due to heterogeneity in the data, meta-analysis was deemed inappropriate for 96% (54/56) of outcomes, which were interpreted qualitatively instead. Nonaggregated analyses of mean differences and CIs between delivery modalities yielded mixed results, with superiority of the digital delivery modality for 18% (10/56) of outcomes, superiority of the face-to-face delivery modality for 5% (3/56) of outcomes, equivalence between delivery modalities for 2% (1/56) of outcomes, and neither superiority of one modality nor equivalence between modalities for 75% (42/56) of outcomes. Consequently, for most outcome measures, no indication of superiority or equivalence regarding the relative efficacy of either delivery modality can be made at this stage. We further meta-analytically compared digital versus face-to-face delivery modalities for attrition (risk ratio 1.03, 95% CI 0.52-2.03; *P=*.93) and number of sessions attended (standardized mean difference –0.11; 95% CI –1.13 to –0.91; *P*=.83), finding no significant differences between modalities, while CIs falling outside the range of the minimal important difference indicate that equivalence cannot be determined at this stage.

**Conclusions:**

Evidence on digital and face-to-face modalities for systemic psychotherapy interventions is largely heterogeneous, limiting conclusions regarding the differential efficacy of digital and face-to-face delivery. Nonaggregated and meta-analytic analyses did not indicate the superiority of either delivery condition. More research is needed to conclude if digital and face-to-face delivery modalities are generally equivalent or if—and in which contexts—one modality is superior to another.

**Trial Registration:**

PROSPERO CRD42022335013; https://tinyurl.com/nprder8h

## Introduction

### Background

Digital delivery of mental health interventions has gained increasing prominence in recent decades. Digital delivery provides solutions to many mental health intervention obstacles, such as limited access [1], geographical distance [2], financial constraints [3], and transportation issues [4]. These factors, and not least the COVID-19 pandemic [5], have contributed to increased delivery of digital modalities of mental health care [6]. One current line of research uses randomized controlled trials (RCTs) to determine the efficacy of digitally delivered family therapies [6] and parenting interventions [7], including but not limited to systemic approaches. This research supports the efficacy of the digital delivery of interventions and finds no evidence for digitally delivered interventions being inferior to face-to-face interventions [8-13]. However, most of the current literature does not compare face-to-face and digitally delivered interventions directly, does not differentiate between the types of digital delivery modalities (eg, therapist-guided videoconferencing and self-guided web-based programs), and does not consider how the modality differentially impacts specific disorders, outcomes, or populations. Furthermore, these studies aggregate findings across broad and diverse contextual definitions of family therapy and parenting interventions instead of applying unified and stringent theory-informed definitions. Although these reviews (eg, McLean et al [6] and Florean et al [7]) do not exclusively focus on a unified and stringent definition, they do provide evidence for systemic approaches in the broad context of family interventions. Many family therapists consider themselves to be systemic therapists, and there is an overlap between family therapy and systemic therapy (ST). However, ST is grounded in a theoretical treatment model, and unlike family therapy, it is not primarily defined by the therapy setting [14]. Thus, ST provides a unified and stringent definition that has practical consequences for research and clinical practice.

ST is a conceptual framework for mental health interventions that focuses on interpersonal relations, interactions, social surroundings and resources, perspectives, and constructions of problems; attempted solutions are appreciated and used as integral parts of the intervention [13,15,16]. Consequently, most family therapy and parenting interventions can be said to contain elements of ST to varying degrees while also potentially including elements from other conceptual frameworks, such as cognitive behavioral therapy and psychoeducation [17]. Conversely, as ST interventions often focus on families as important resources requiring incorporation into intervention delivery [18], they can, but need not be, similar to other family therapy or parenting interventions in terms of including other family members in their setting. The efficacy of ST as a face-to-face intervention is well documented in various reviews and meta-analyses for a range of psychological disorders for youth and adult populations [13,15-17,19]. In line with developments for other psychotherapeutic interventions, the implementation of digital delivery modalities of ST has rapidly increased in recent years [20-22]. The implementation of videoconferencing is particularly promising, and the experiences of practitioners implementing this delivery modality are overall positive [23]. Despite such indications, there are gaps in the literature [20,24]. There is an urgent need to provide an evidence base for practitioners to understand how face-to-face and digital delivery of the same ST intervention compare (compare McLean et al [6], Florean et al [7], and Fairburn and Patel [25]).

The need for greater granularity regarding the comparative efficacy of digital and face-to-face delivery modalities is in line with the more general requirement to identify “conditions under which systemic therapy works best” [16]. Thus, we simultaneously address both the need for a systematic review and meta-analysis that investigates (1) how various types of ST interventions, delivered in their traditional face-to-face form, compare to the same ST intervention delivered digitally with regard to their efficacy and (2) delivery modality as key contextual factors that modulate ST intervention outcomes.

### Research Questions

We conducted a systematic review and meta-analysis to address the following research questions: (1) What are the characteristics, quality, and resulting evidence from published RCTs comparing the efficacy of ST interventions using digital treatment modalities with ST interventions using face-to-face treatment modalities across settings, populations, and outcomes? (2) If a meta-analysis of RCTs comparing ST interventions in digital and face-to-face modalities can be conducted, what is the difference in delivery modalities for mental disorder outcomes?

## Methods

### Overview

This systematic review followed reporting guidelines as laid out in the PRISMA (Preferred Reporting Items for Systematic Reviews and Meta-Analyses) 2020 statement (Multimedia Appendix 1 2020 checklist) [26]. We aimed to identify, evaluate, and synthesize findings from RCTs comparing digital and face-to-face delivery modalities for ST interventions across settings, target populations, and outcomes. This systematic review was preregistered (PROSPERO ID: CRD42022335013), with no deviation from the registered protocol.

### Inclusion and Exclusion Criteria

We applied the following inclusion and exclusion criteria for articles to be considered for our systematic review within the following categories: (1) study design, (2) participants, (3) interventions, (4) digital delivery and face-to-face delivery modalities, (5) outcomes, and (6) article type.

RCTs of any type were included (eg, parallel, cluster, and quasi-RCTs). No minimum number of participants was prespecified.

Only those articles were deemed eligible for inclusion that involved individual participants of all ages and genders (1) with one or more diagnoses of a mental disorder, (2) identified as at risk in one or more mental or behavioral health domains, or (3) adversely affected by salient circumstances (eg, belonging to a structurally disadvantaged community and caring for a family member). Articles involving participants at the system level (eg, families) were also included as long as 1 member of the system fulfilled at least one of the aforementioned criteria. No further exclusion criteria were applied to increase the number of eligible studies.

We only included articles reporting on interventions delivered via at least 2 distinct delivery modalities: digital delivery and face-to-face delivery. Digital delivery was defined as exclusively relying on an external electronic medium (eg, videoconferencing hardware and software, telephone, and synchronous and asynchronous text-based conversations) for intervention delivery. While this allowed heterogeneity of digital delivery modalities across some dimensions (eg, synchronicity and extent of interaction with mental health practitioners), it kept other dimensions stable across all digital delivery modalities (use of electronic medium and no mental health practitioner physically copresent with recipients of intervention). Face-to-face delivery was defined as exclusively relying on in-person intervention delivery. This allowed for the use of external tools or media to supplement the intervention while specifying that intervention delivery had to occur solely between people physically present in the same physical space (eg, a clinician’s office).

To increase the number of eligible studies, any relevant mental, behavioral, or somatic health efficacy; adherence and attrition; and further outcomes were included in this review. No a priori categorization of outcomes was applied. Efficacy outcomes were defined as any outcomes pertinent to improvement in relevant mental, behavioral, and somatic health domains. *Further outcomes* were defined as any outcomes not directly pertinent to improvement in relevant health domains (eg, subjective satisfaction rating of program delivery).

There were no restrictions imposed on the article type in terms of language or date. Articles published in peer-reviewed journals as full-text articles and other types of peer-reviewed full-text articles were included. Unpublished data (eg, dissertations), as well as study protocols, clinical trial registries, and non–peer-reviewed articles, were excluded.

### Intervention Inclusion Criteria

Only articles reporting on ST interventions delivered via both digital and face-to-face delivery modalities were included. ST was operationalized based on 4 adapted definition criteria of ST [13], which were applied to the descriptions of the identified constituent primary parts (CPPs; eg, core sessions) of interventions. The definition criteria were derived from the following definition of ST (referred to as *systemic therapy*) with our adjustments in square brackets [13]:

We use systemic/systems-oriented therapy/therapies (ST) as a general term for a major therapeutic orientation that can be distinguished from other major approaches (e.g., CBT or psychodynamic therapy). We define systemic therapy as a form of psychotherapy that (1) perceives behavior and mental symptoms within the context of the social systems people live in; (2) focuses on interpersonal relations and interactions, social constructions of realities, and[/or] the recursive causality between symptoms and interactions; (3) includes family members and[/or] other important persons (e.g., teachers, friends, professional helpers) directly or indirectly through systemic questioning, hypothesizing, and specific interventions; and (4) appreciates and utilizes clients’ perspectives on problems, resources, and[/or] preferred solutions.

For each intervention, the appropriate unit of primary intervention delivery (as opposed to parts of the intervention labeled *supplementary*, *additional*, or *optional*, etc) was determined following the study’s authors (eg, core sessions, core components, etc.). The total number of the intervention’s CPPs was determined. If the intervention description did not afford to determine an appropriate unit of intervention delivery, the total number of CPPs was defined as 1.

For each description of content for any CPP, we assessed if it met any of our definition criteria of ST. Notably, any particular definition criterion could, in theory, be met by a set of CPP descriptions >1, allowing different content descriptions to meet the same criterion. This allowed a range of different interventions to be classified as being similar in kind (ie, being ST interventions), reflecting the integrative nature of clinical ST practice [27,28], rather than requiring all interventions to have identical CPPs.

All definition criteria of ST needed to be met by the respective CPPs, with at least 2 of the 4 definition criteria being met completely by all CPPs. If any of the definition criteria of ST were not completely met, at most 2 of the 4 definition criteria needed to be met by a minimum of at least 50% of all CPPs (or, in the case of uneven numbers of CPPs, the closest possible number below the theoretical half point). The same CPP could satisfy >1 criterion. Definition criterion (1) could additionally be met by the relevant conceptual background provided.

Textbox 1 illustrates an example for 1 trial included in our study. Our approach used to screen trials against inclusion criteria for all trials is detailed in Multimedia Appendix 2 [13,29-32].

**Identified constituent primary parts (CPPs) of 1 trial against systemic therapy (ST) definition criteria for Behavioral Family Systems Therapy for Diabetes (BFST-D) intervention [33].**

**Number and unit of CPPs and 4 primary intervention components**

1. Perceives behavior and mental symptoms within the context of the social systems people live in
  Family functioning and maladaptive parent-child interactions were identified as central barriers to diabetes treatment adherence.
2. Focuses on interpersonal relations and interactions, social constructions of realities, or the recursive causality between symptoms and interactions: 3 of 4 CPPs
  CPP1: family-problem solving (as a family, defining the problem, generating solutions, making decisions, implementing and monitoring results, and refining ineffective solutions)
  CPP2: communication training (instruction, feedback, modeling, and rehearsal of approaches toward improving maladaptive communicative patterns)
  CPP4: family restructuring (functional and structural approaches toward changing maladaptive or ineffective family system patterns and characteristics, such as weak parental coalitions or cross-generational coalitions).
3. Includes family members and other important persons (eg, teachers, friends, professional helpers) directly or indirectly through systemic questioning, hypothesizing, and specific interventions
  All CPPs are delivered to the caregiver-adolescent dyad.
4. Appreciates and uses clients’ perspectives on problems, resources, and preferred solutions: 2 of 4 CPPs are as follows:
  CPP1 and CPP3: cognitive restructuring (addressing beliefs, attitudes, and attributions that could negatively affect effective interactions).

### Search Strategy

#### Database Search

The following databases were searched from the earliest available date until March 15, 2022: PubMed, Embase (via Ovid), Cochrane CENTRAL (via Ovid), CINAHL (via EBSCO), PsycINFO (via EBSCO), and PSYNDEX (via EBSCO). The search strategy followed the guideline from Bramer et al [34], and the original search string was developed for use in PubMed and adapted manually for all other databases. The search strings contained a set of the following: (1) 1 compiled term for intervention type, (2) 1 compiled specificity term to define the target domain, and (3) 1 compiled delivery modality term. For the identification of RCT study design, we used the RobotSearch AI RCT highly sensitive filter [35], which provides high sensitivity in identifying RCTs [35-37]. Multimedia Appendix 3 [8,13,15,38-46] provides the full search strings, references of the published sources upon which we based our search terms, and explanations of the logical structure. On March 17, 2022, we conducted complementary searches in ClinicalTrials.gov, the International Clinical Trials Registry Platform, and the EU Clinical Trials Register (all via the respective standard website interface). On May 4, 2022, backward citation screening (articles cited) was conducted on identified relevant reviews and meta-analyses (Multimedia Appendix 4 [9,10,12,13,15,47-53]). On May 10, 2022, forward citation screening (articles citing included articles) was conducted via ISI Web of Science, backward citation screening on included articles was conducted via PubMed, and a “related articles” screening was conducted on included articles (first 20 articles listed as related articles) via PubMed.

#### Article Selection and Screening

Duplicates were removed using Automated Systematic Search Deduplicator [54]. The resulting list of search results was split into 2 sets (sorting by title in EndNote [version 20; Clarivate] and allocating alternating sets of approximately 100 consecutive references; number of articles=1816 and 1817) that were each assigned to 2 pairs of authors (PE and JB; MB and JB), with each author independently screening titles and abstracts using manualized standard operating procedures that we developed accommodating our inclusion and exclusion criteria. All discrepancies at the title and abstract screening stage were resolved through discussion between all 3 involved authors (PE, MB, and JB). PE and MB independently screened at the full-text stage. Discrepancies at this stage were resolved through discussion between PE and MB. GM was consulted as an independent author of this study in cases of persisting discrepancies after discussions at any stage. Because on several occasions a trial underlying an included article contributed data to >1 included article, article-trial correspondence was determined either via trial registration numbers (for 7 articles) or via references to articles on the same trial, unique trial names, as well as overlap in article authors, article dates, sample sizes and characteristics, interventions, and procedures (for 5 articles).

### Data Extraction

Data were independently extracted by PE and MB using the Cochrane data collection form [55] for intervention reviews (Multimedia Appendix 5 [29,30,33,56-59]). Discrepancies were resolved through discussion.

### Risk of Bias

The authors (PE and MB) independently assessed the risk of bias (RoB) using the revised Cochrane Risk of Bias (version 2) tool for randomized trials [60] and following the process outlined in chapter 8 of the Cochrane handbook [55]. Discrepancies were resolved through discussion. RoB figures were created using *robvis* [61].

### Data Synthesis and Analysis

We used RevMan (version 5.4) [62] to create forest plots and calculate, depending on the variable type and type of analysis, mean differences (MDs), standardized MDs, risk ratios, and associated CIs at the 95% level. Where possible, we calculated missing SDs using the Cochrane collaboration RevMan calculator [62]. We conducted meta-analyses only in cases of acceptable levels of clinical and methodological diversity and where sufficient data were available [63]. We conducted meta-analyses using random effects models as we expected heterogeneity across articles to be high. Statistical heterogeneity was assessed using the *I*^2^ statistic. MDs and CIs were calculated where meta-analytic analysis was not possible, but nonaggregated analysis was still deemed appropriate. This was to increase qualitative comparability across studies with nonnegligible heterogeneity in statistical approaches and reported results. All relevant reported results are listed in Multimedia Appendix 6 [29,30,33,56-59,64-68]. We grouped MDs of outcomes according to their CIs into (1) fully located within the range from –0.20 to 0.20 (*equivalence*), (2) fully located outside this range (*superiority*), or (3) partially located within and partially located outside this range (*neither equivalence nor superiority*). In the absence of an established a priori minimal important difference, we accept Cohen *d* cutoff of 0.2 (small effect) as the defined range from –0.20 to 0.20 to indicate equivalence [69]. We interpreted a CI of an MD falling within, but not exceeding, this range as evidence for equivalence, a CI of an MD falling entirely outside this range as evidence for nonequivalence or superiority of one delivery modality, and a CI falling within and exceeding this range as insufficient evidence to determine equivalence or nonequivalence on the respective measure. Where calculating MDs and CIs was impossible due to missing information, reported results on differences between digital and face-to-face delivery modalities were provided (Multimedia Appendix 6). Study authors were contacted to provide missing information. A funnel plot was not created because we identified <10 trials [70].

## Results

### Search Results

**Figure 1:**
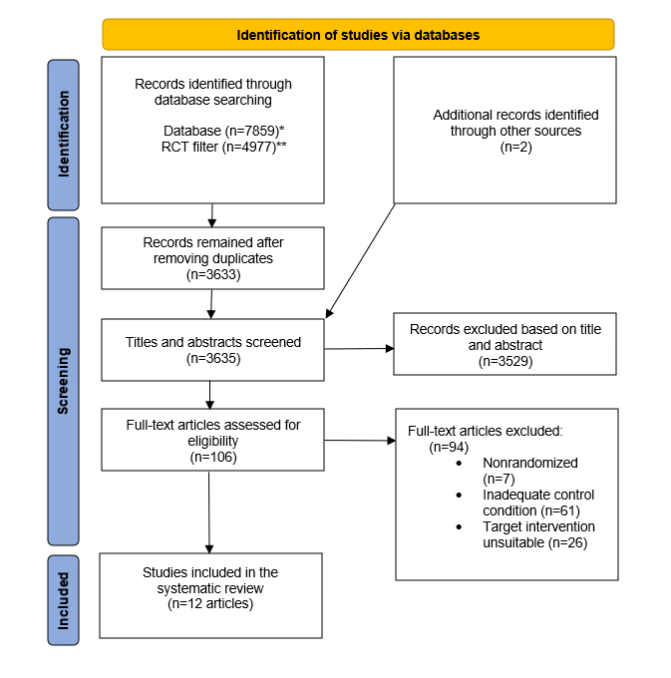
PRISMA (Preferred Reporting Items for Systematic Reviews and Meta-Analyses) flowchart of the articles included in the systematic review. RCT: randomized controlled trial. *Results from CINAHL (EBSCO): n=432; PSYNDEX (EBSCO): n=53; PsycINFO (EBSCO): n=505; Embase (Ovid): n=2177; Cochrane (Wiley): n=533; PubMed: n=4159; **RobotSearch AI, Marshall et al [35].

A flowchart of our search and screening process is depicted in Figure 1 [35]. The primary search for this study retrieved 4977 references and 3633 after removal of duplicates. Additional references (n=2) were identified through complimentary searches. The resulting 3635 references were screened at the title and abstract level. On the basis of our inclusion and exclusion criteria, 97.1% (3529/3635) articles were excluded during the title and abstract screening, and 2.9% (106/3635) articles were screened at the full-text screening stage. Overall, 0.3% (12/3633) of the initially identified articles reporting on 4 trials were included in this systematic review. Some (3/12, 25%) of the articles reporting on 2 trials [29,56,57] are only reported in Table 1 and Multimedia Appendix 6, as they could not be included in further meta-analytic aggregation or discrete analysis of MDs and CIs.

**Table 1:** Characteristics of included studies (N=754)^a^.

Trial and articles			Trial sample size (% of N; F2F^b^; DD^c^)		Youth age (y), mean (SD)		Male, n (%)		Feature		DI^d^		F2FI^e^		Sessions, mean (SD)
**BFST-D^f^**															
	Duke et al [29], 2016	90 (11.9%; 46; 44)		15.02 (1.75)		55 (61.1)		HbA_1c_^g^ >9%		Videoconferencing		Clinic		5.8 (3.3)	
	Harris et al [58], 2015	90 (11.9%; 46; 44)		14.9^h^ (1.7)		55 (61.1)^h^		HbA_1c_ >9%		Video conferencing		Clinic		5.8 (3.3)	
	Freeman et al [33], 2013	92 (12.2%; 45; 47)		15.1^i^		42 (45.7)^h^		HbA_1c_ >9%		Videoconferencing		Clinic		—^j^	
	Riley et al [57], 2015	82 (10.9%)^k^		14.1		55 (61.1)		HbA_1c_ >9%		Videoconferencing		Clinic		6.3 (3.4)	
	**PAAS^l^**														
	Murry et al [56], 2019	421 (55.8%; 141; 141; CC^m^: 136)		—		195^h^ (46)		PRB^n^		II^o^		GS^p^		—	
	Murry et al [59], 2019	412 (54.6%; 137; 138; CC: 137)		11.0		191^h^ (46)		PRB		II		GS		—	
	Murry et al [30], 2018	412 (54.6%; 137; 138; CC: 137)		11.4		191 (46.4)		PRB		II		GS		4.0 (3.0)	
	**F-PST^q^**														
	Kurowski et al [65], 2020	150 (19.9%; 34; 56; SG^r^: 60)		16.5 (1.1)		96 (64)		TBI^s^		Videoconferencing+OP^t,u^		Clinic		6.3 (2.6)	
	Wade et al [66], 2019	150 (19.9%; 34; 56; SG: 60)		16.5 (1.1)		96 (64)		TBI		Videoconferencing+OP^u^		Clinic		6.3 (2.6)	
	Wade et al [68],2019	150 (19.9%; 34; 56; SG: 60)		16.5 (1.1)		96 (64)		TBI		Videoconferencing+OP^u^		Clinic		6.3 (2.6)	
	Wade et al [67],2019	150 (19.9%; 34; 56; SG: 60)		15.5 (1.5)^v^		96 (64.0)		TBI		Videoconferencing+OP^u^		Clinic		6.3 (2.6)	
	**SUCCEAT^w^**														
	Truttmann et al [64], 2020	102 (13.5%; 50; 52)		14.9 (1.9)		9 (8.9)		AN^x^		OP		GWS^y^		6.5 (2.0)	

^a^Sum of n at the trial level. In case of inconsistencies in reported n across individual articles for each trial, the mode n was selected.

^b^F2F: face-to-face delivery.

^c^DD: therapist-guided digital delivery.

^d^DI: digital implementation.

^e^F2FI: face-to-face implementation.

^f^BFST-D: Behavioral Family Systems Therapy for Diabetes.

^g^HbA_1C_: glycated hemoglobin.

^h^Calculated based on percentages and overall sample size provided.

^i^Calculated based on mean ages, sample sizes, and SDs provided.

^j^Not available.

^k^Only participants who completed the Child Depression Inventory measure (only those aged <18 y eligible).

^l^PAAS: Pathways for African American Success.

^m^CC: control condition.

^n^PRB: potential risk behavior.

^o^II: interactive interface.

^p^GS: group session.

^q^F-PST: Family Problem-Solving Therapy.

^r^SG: self-guided digital delivery.

^s^TBI: traumatic brain injury.

^t^OP: online program.

^u^Two distinct digital delivery conditions: (1) videoconferencing+web-based program and (2) online program.

^v^Most likely a clerical mistake in reporting.

^w^SUCCEAT: Supporting Carers of Children and Adolescents with Eating Disorders in Austria.

^x^AN: anorexia nervosa.

^y^GWS: group workshop.

### Characteristics of Included Articles

#### Number of Trials, Articles, and Sample Size

A total of 12 articles reporting on 4 trials were identified. One trial was reported on by a single article [64] (Supporting Carers of Children and Adolescents with Eating Disorders in Austria [SUCCEAT] trial), while the remaining 92% (11/12) of articles reported on a total of 3 trials. Of all 12 articles, 58% (n=7) of articles provided trial registration numbers, allowing for formally confirmed identification of the reported trial.

For the purposes of this study, the 4 trials are abbreviated according to their respective intervention names as follows: Behavioral Family Systems Therapy-Diabetes (BFST-D) trial [29,33,57,58], Family Problem-Solving Therapy (F-PST) trial [65-68], Pathways for African American Success (PAAS) trial [30,56,59], and the SUCCEAT trial [64].

As articles reporting on the BFST-D trial and articles reporting on the PAAS trial varied with regard to sample sizes (Table 1 and Multimedia Appendix 6), the total number of randomized participants across all trials could not be unambiguously determined. Given that for both trials, only 1 article each reported a different sample size as compared to all other articles reporting on the same trial, we reported the mode sample size for the total number of participants randomized to all conditions in all trials, including a control condition (N=754), and for the total number of participants randomized to face-to-face and digital delivery conditions (n=617). The same principle was applied for all reported participant characteristics, which varied in terms of proportions and absolute numbers depending on variation in reported sample sizes.

#### Participants

Some (4/12, 33%) articles reported on interventions delivered to youths with poorly controlled type 1 diabetes and their parents (BFST-D trial), 25% (n=3) articles reported on interventions delivered to parents, siblings, and youths identified as being at disproportionate risk for contracting HIV and sexually transmitted infections, teen parenthood, and substance use (PAAS trial). Moreover, 33% (4/12) articles reported on interventions delivered to youths with traumatic brain injury and their parents and other family members (F-PST trial), and 8% (1/12) articles reported on interventions delivered to parents of youths with anorexia nervosa (SUCCEAT trial). As there is inconsistent terminology between and within articles, the term *parent* is used here and throughout this paper to refer to parents, grandparents, legal guardians, and caregivers included in the respective articles. All identified interventions either involved families in intervention delivery (BFST-D, PAAS, and F-PST trials) or in terms of interpersonal target outcomes (SUCCEAT trial). Additional participant information is provided in Table 1 and Multimedia Appendix 7 [29,30,33,56-59,64-68]. Participant characteristics were reported to be similar across conditions in all articles, with the exception of the F-PST trial, which included a lower proportion of White participants in the face-to-face delivery condition, probably linked to quasi-randomization involving place of residence in the allocation procedure.

#### Face-to-Face and Digital Interventions and Delivery Format

Interventions were provided by health professionals in 75% (9/12) of articles (BFST-D, F-PST, and SUCCEAT trials) and by trained community leaders in 25% (3/12) articles (PAAS trial). Intervention content was similar across delivery modalities for all trials, with all articles explicitly mentioning the same intervention being delivered across conditions and all CPPs of interventions being delivered in both conditions (Table 1 and Multimedia Appendix 2).

All therapist-guided digital delivery was provided via videoconferencing software. Self-guided intervention delivery was provided via access to web-based modules for 33% (4/12) of the articles (F-PST trial) and for 25% (3/12) of the articles (PAAS trial) via an interactive interface visually representing users through avatars in a virtual environment. Face-to-face delivery was reported as individual in-clinic sessions for 67% (8/12) of the articles (BFST-D and F-PST trials), as group sessions at community centers for 25% (3/12) of the articles (PAAS trial), and in-clinic workshops for multiple individuals for 8% (1/12) of the articles (SUCCEAT trial).

#### Outcomes

Across all 12 articles, 56 outcome measures (ie, dependent variables) were reported, including cases of parent-report and youth-report measures of the same construct. Primary outcomes (8/56, 14%), 71% (40/56) secondary outcomes, and 14% (8/56) further, dropout, or attrition outcomes were identified. We have provided an overview of all identified outcomes in Multimedia Appendix 8 [29,33,56-59,64-68].

We summarized and structured data along three domains: (1) outcome type (ie, youth, parent, family functioning, and dropout, attrition, or further), (2) delivery modality type (face-to-face, therapist-guided digital, and self-guided digital), and (3) measurement time point (postintervention test and follow up test) to reduce clinical and methodological diversity within outcome data categories. We provide a more detailed overview of delivery modality types (eg, videoconferencing or in-clinic sessions) in Table 1, an overview of outcome measures (ie, dependent variables) in Multimedia Appendix 8, and measurement time points in Multimedia Appendix 6.

The application of these 3 domains to the categorization of data yielded 16 potential outcome categories: youth (Multimedia Appendix 9 [58,59,64-67]); parent (Multimedia Appendix 9); family functioning (Multimedia Appendix 10 [59]); and adherence, attrition, and further outcomes (Multimedia Appendix 11 [30,33,62,65,68]) for either therapist-guided digital versus face-to-face delivery (Multimedia Appendix 9) or self-guided digital versus face-to-face delivery modalities (Multimedia Appendices 9 and 10) at either posttest (Multimedia Appendices 9 and 10) or follow-up test (Multimedia Appendix 11) time points.

### Risk of Bias

#### Overview

**Figure 2:**
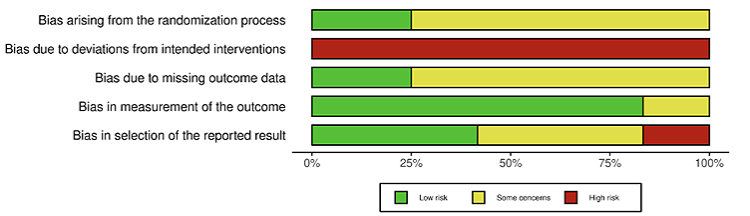
Total proportions of articles’ risk of bias assessments by assessment domain.

**Figure 3:**
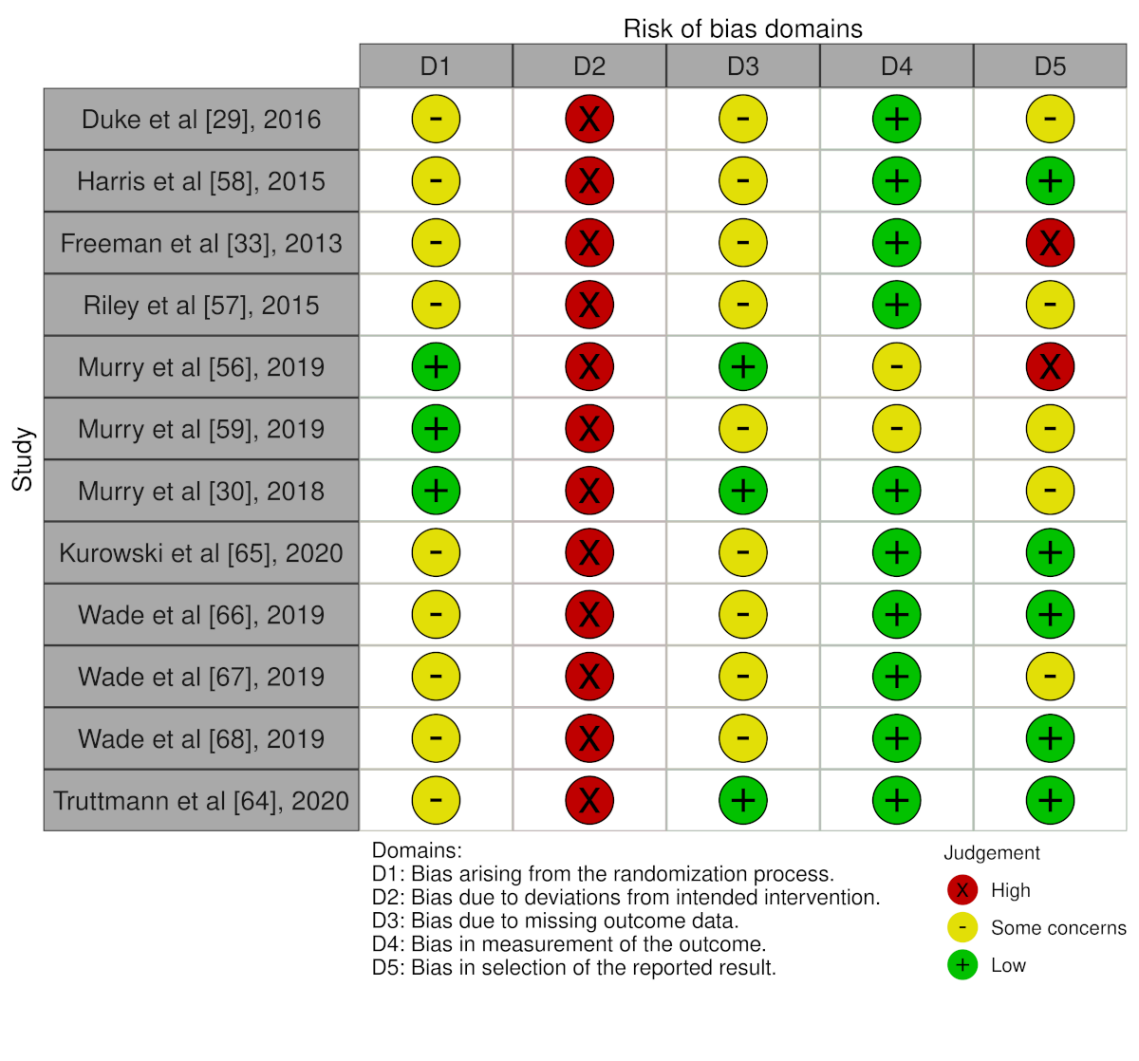
Risk of bias assessments of all included articles. D1: bias arising from the randomization process; D2: bias due to deviations from intended intervention; D3: bias due to missing outcome data; D4: bias in measurement of the outcome; D5: bias in selection of the reported result.

The 12 articles were rated as high risk for allocation concealment. For sequence generation, 75% (9/12) articles were rated as having some concerns. For randomization, 2 of the 4 trials used quasi-randomization. Some (9/12, 75%) articles were rated as having some concerns in terms of missing data. Overall proportions of RoB levels across articles are presented in Figure 2; individual RoB assessments at the article level are presented in Figure 3 [29,30,33,56-59,64-68]. Moreover, 92% (11/12) articles reported on 3 trials, with inconsistent reporting at times, which constituted a challenge for assessing the RoB, particularly for the domain of selective outcome reporting, as multiple articles reporting on the same trial were found to simultaneously overlap in some reported outcomes and diverge for other outcomes (eg, inconsistent reporting of the Diabetes Family Conflict Scale across articles reporting on the BFST-D trial).

#### Sequence Generation

Of all 12 articles, 8% (n=1) reported a quasi-randomization temporal sequencing design (SUCCEAT trial); 33% (n=4) reported a quasi-randomized, multicenter RCT design (F-PST trial), in which participants were unequally allocated to the digital and face-to-face delivery conditions based on their place of residence (all participants who lived outside a 40-kilometer radius from the site were randomly allocated to the digital delivery conditions, and participants within the radius were randomly allocated to all conditions, favoring the face-to-face delivery condition by a ratio of 2:1); and 58% (n=7) reported randomized controlled trials (BFST-D and PAAS trials). Of those 7 articles, 57% (n=4) reported block randomization without providing information on allocation sequence concealment, increasing the RoB from sequence generation (BFST-D trial). Overall, 25% (3/12) of the included articles were rated low RoB arising from the randomization, while 75% (9/12) were rated as giving rise to some concerns.

#### Allocation Concealment

The question of adequate allocation blinding procedures (for participants and intervention providers) is a common concern in psychotherapy outcome research [71]. This also applies to the articles included in this review. Given the absence of a universally agreed-upon alternative approach [71], all articles were rated as high RoB in this domain.

#### Handling of Incomplete Data

For any of the 12 articles, not all outcome data were available. Some (2/12, 17%) of the articles provided evidence of data missing at random [56] or treated attrition as an outcome variable [30] and were rated as having low RoB. Most (9/12, 75%) articles did not adequately analyze if missing outcome data did not potentially bias the results and were rated as giving rise to some concerns.

#### Outcome Assessment

RoB arising from the measurement of the outcome variables was rated *low risk* for 83% (10/12) of the articles and with *some concerns* for 17% (2/12) of the articles that used a large proportion of outcome measures for which judgments regarding validity and reliability could not be made [56,59] (Multimedia Appendix 8).

#### Selective Reporting

As indicated earlier, RoB from selective reporting was challenging to assess given the interaction of multiple articles reporting on the same trial and inconsistencies across articles on the same trials. This was further exacerbated in the case of a trial for which no preregistration could be identified (PAAS trial) and of an article exclusively reporting on unregistered outcomes of a preregistered trial [33]. Some (5/12, 42%) articles were rated as giving rise to some concerns, 9% (5/12) were rated as having low RoB, and 17% (2/12) of the articles were rated as having high RoB.

### Appropriateness of a Meta-Analysis to Determine the Efficacy of Delivery Modalities, Adherence, and Attrition

**Figure 4:**
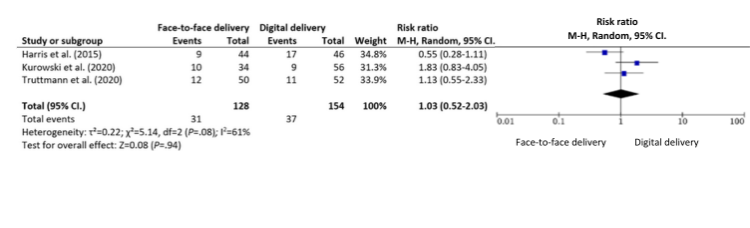
Meta-analysis of number of sessions attended in face-to-face versus digital delivery modality comparison.

**Figure 5:**
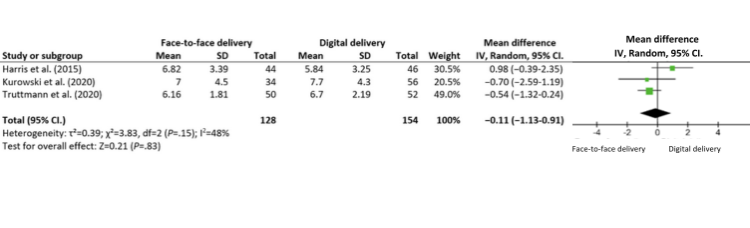
Meta-analysis of drop-out (event) risk ratio in face-to-face versus digital delivery modality comparison.

Meta-analytic aggregation was deemed inappropriate for all delivery modality comparisons of youth, parent, and family functioning outcomes due to the relatively low number of trials within the respective outcome categories, combined with the presence of substantial clinical and methodological diversity across trials [63]. Meta-analytic aggregation of the number of sessions attended and attrition was conducted because heterogeneity was lower and the number of eligible trials was higher for these outcomes as compared to the other outcome measures reported and included (Figure 4 [58,64,65] and Figure 5 [58,64,65]). The substantial clinical and methodological diversity across trials remains problematic for drawing meaningful conclusions using meta-analytic aggregations. However, a preliminary quantitative synthesis was included as it still serves as a starting point for discussion and future research.

### Nonaggregated Analyses for Face-to-Face and Digital Delivery Modalities

Of all 56 reported outcomes (ie, dependent variables) across all trials and articles, 25% (n=14) outcomes from 2 trials (13/14, 93% in the F-PST trial and 1/14, 7% in the SUCCEAT trial) showed MDs with corresponding CIs falling either (1) fully within our predefined range of minimal important difference of –0.20 to 0.20 or (2) fully outside of this range. These outcomes are reported in Table 2 and Multimedia Appendix 12. All remaining 75% (42/56) outcomes showed MDs with corresponding CIs partially located within and partially located outside this range. Among these, 4% (2/56) further outcomes, 2% (1/56) family functioning outcome, and 2% (1/56) youth outcome were reported as substantially different between delivery modalities in the original articles while still including our range for the minimal important difference. Multimedia Appendix 9 shows a full list of all MDs and CIs of outcomes.

**Table 2:** Outcomes with substantial differences (95% CI excluding 0) or equivalence across all trials (N=252; sum of n at the trial level).

Outcome type, postintervention test and follow-up test, trial, and article				Outcome	Type of DD^a^	F2F^b^ delivery, n	F2F delivery, mean (SD)	DD, n	DD, mean (SD)	Mean difference (95% CI)	Directionality
**Youth**											
	**Postintervention test**										
		**F-PST^c^; Wade et al [67], 2019**									
			PedsQL^d^, parent report		TG^e^	22	62 (15.71)	43	70.9 (18.49)	–8.90 (–17.48 to –0.32)	Favors DD
			PedsQL, parent report		SG^f^	22	62 (15.71)	51	71.7 (18.85)	–9.70 (–18.06 to –1.34)	Favors DD
	**Follow-up test**										
		**F-PST; Wade et al [67], 2019**									
			HBI^g^ somatic, youth report		TG	22	9.8 (4.92)	44	6.6 (5.90)	3.20 (0.50 to 5.90)	Favors DD
			HBI cognitive, parent report		SG	22	19.1 (8.58)	48	12.3 (9.91)	6.80 (2.25 to 11.35)	Favors DD
			HBI somatic, youth report		SG	22	9.8 (4.92)	48	6.8 (5.89)	3.00 (0.35 to 5.65)	Favors DD
			PedsQL, parent report		SG	22	67 (16.18)	48	76.6 (18.98)	–9.60 (–18.23 to –0.97)	Favors DD
**Parent**											
	**Postintervention test**										
		**F-PST; Wade et al [66], 2019**									
			CES-D^h^		TG	22	15.1 (1.81)	43	12.8 (1.53)	2.30 (1.42 to 3.18)	Favors DD
			BSI^i^		TG	22	56.1 (2.46)	43	53.3 (2.02)	2.80 (1.61 to 3.99)	Favors DD
			CES-D		SG	22	15.1 (1.81)	51	13.5 (1.42)	1.60 (0.75 to 2.45)	Favors DD
			BSI		SG	22	56.1 (2.46)	51	54.7 (1.88)	1.40 (0.25 to 2.55)	Favors DD
		**SUCCEAT^j^; Truttmann et al [64], 2020**									
			SCL^k^-90-R		TG	48	0.24 (0.26)	46	0.31 (0.36)	–0.07 (–0.20 to 0.06)	Equivalence
	**Follow-up test**										
		**F-PST; Wade et al [66], 2019**									
			BSI		TG	22	51.7 (2.42)	44	53.3 (2.02)	–1.60 (–2.77 to –0.43)	Favors F2F delivery
			CES-D		SG	22	11.8 (2.03)	48	13.2 (1.58)	−1.40 (−2.36 to −0.44)	Favors F2F delivery
		**Wade et al [68], 2019**									
			P-PE^l^		TG	22	9.0 (1.4)	43	7.8 (2.1)	1.20 (0.34 to 2.06)	Favors F2F delivery

^a^DD: digital delivery.

^b^F2F: face-to-face.

^c^F-PST: Family Problem-Solving Therapy.

^d^PedsQL: Pediatric Quality of Life Inventory.

^e^TG: therapist guided.

^f^SG: self-guided.

^g^HBI: Health and Behaviour Inventory.

^h^CES-D: Center for Epidemiological Studies Depression Scale.

^i^BSI: Brief Symptom Inventory.

^j^SUCCEAT: Supporting Carers of Children and Adolescents with Eating Disorders in Austria.

^k^SCL: Symptom Checklist 90-Revised Global Severity Index.

^l^P-PE: Parent Rated Program Evaluation.

Of the 14 outcomes falling fully within or fully outside the minimal important difference range, superiority of the digital delivery condition was found for 71% (n=10) outcomes. Of these 10 outcomes, 60% (n=6) were youth outcomes and 40% (n=4) were parent outcomes. The quality of life of youths, reported by parents (Pediatric Quality of Life Inventory, parent report) at post intervention test, was substantially higher for both therapist-guided and self-guided digital delivery modalities compared to face-to-face delivery modalities and remained substantially higher for the self-guided digital delivery condition at follow-up. Somatic symptoms of youths, reported by them (Health and Behaviour Inventory somatic, youth report) at follow-up, were substantially higher in the face-to-face delivery condition compared to both therapist-guided and self-guided digital delivery conditions. In addition, cognitive symptoms of youths, reported by parents (Health and Behaviour Inventory cognitive, parent report) at follow-up, were substantially higher in the face-to-face delivery condition compared to the self-guided digital delivery condition. Depressive symptoms of parents (Center for Epidemiological Studies Depression Scale) at post intervention test were substantially higher in the face-to-face delivery condition compared to both therapist-guided and self-guided digital delivery conditions. Similarly, mental symptoms of parents (Brief Symptom Inventor) at post intervention measurement were substantially higher in the face-to-face delivery condition compared to both therapist-guided and self-guided digital delivery conditions.

Superiority of the face-to-face delivery condition was found for 3 outcomes. All of these outcomes were parent outcomes from the F-PST trial at follow-up. In contrast to comparisons at post intervention measurement, depressive symptoms of parents (Center for Epidemiological Studies Depression Scale) at follow-up were substantially higher in the self-guided digital delivery condition compared to the face-to-face delivery condition. Similarly, contrasting with comparisons at post intervention measurement, mental symptoms of parents (Brief Symptom Inventory) at follow-up were substantially higher in the therapist-guided digital delivery condition compared to the face-to-face delivery condition. Program evaluation by parents was more favorable in the face-to-face delivery condition compared to the therapist-guided digital delivery condition.

Only 2% (1/56) of outcomes fell fully within the minimal important difference range. In the SUCCEAT trial, psychopathological symptoms of parents (Symptom Checklist 90-Revised Global Severity Index) at post intervention test were equal between therapist-guided digital and face-to-face delivery conditions.

### Meta-Analytic Aggregation of Adherence and Attrition

A total of 9 articles reporting on all 4 trials (BFST-D trial [29,57,58], PAAS trial [59], F-PST trial, and SUCCEAT trial) reported on the number of sessions attended, and all (11/12, 92%) articles, except for 1 (PAAS trial [57]), reporting on all trials reported on attrition. The number of sessions attended and attrition were meta-analyzed, and the meta-analytic aggregations are reported in Figures 4 and 5. The heterogeneity between trials was moderate (*I*^2^=48% for number of sessions attended) to high (*I*^2^=61% for attrition), and given the low number of included trials, this heterogeneity estimate might be inaccurate [72], and conclusions from the meta-analyses should thus be interpreted with caution.

## Discussion

### Principal Findings

We identified 12 articles reporting on 4 trials from which data were extracted (Multimedia Appendix 5). Of note, despite broad inclusion criteria for participants, participants included only youths or parents of youths. Recipients of interventions were youths with poor diabetic control, traumatic brain injuries, increased risk behavior likelihood, and parents of youths with anorexia nervosa. A total of 56 relevant outcomes were identified. Two trials provided digital intervention delivery via videoconferencing, one via an interactive graphic interface, and the other via a web-based program. RoB judgments varied substantially across domains and articles, with largely *some concerns* judgments for randomization, missing outcome data, and selective reporting. Applying a minimal important difference criterion to indicate equivalence or superiority, nonaggregated analyses of MDs and CIs between delivery modalities yielded varying types of comparative outcomes across the included studies: superiority of the digital delivery modality and superiority of the face-to-face delivery modality (CIs falling fully outside the range of the minimal important difference) and equivalence between delivery modalities (CIs falling fully inside the range of the minimal important difference), with most comparative outcomes, indicating neither superiority of one modality nor equivalence between delivery modalities (CIs falling within and outside the range of the minimal important difference). Overall, our analyses did not reveal any strong evidence for the superiority of face-to-face compared to digital delivery conditions and might tentatively instead indicate a higher proportion of favorable effects of digital delivery modalities for certain outcomes and certain contexts. Due to the qualitative nature of this analysis, the limited number of studies included in this review, and the large heterogeneity between studies, results need to be interpreted with caution. In addition, more research is needed to draw meaningful conclusions on the comparative efficacy of digital versus face-to-face delivery modalities of ST interventions.

We found a limited and insufficient amount of evidence to meta-analytically estimate the comparative efficacy of digital versus face-to-face delivery modalities of ST interventions with regard to most psychological, somatic, and behavioral parent and youth outcomes, as well as family functioning and further outcomes at either postintervention test or follow-up test. Meta-analytic analysis was deemed appropriate only for attrition and the number of sessions attended. We compared digital versus face-to-face delivery modalities for attrition and the number of sessions attended, showing no evidence for the superiority of either delivery modality in these domains.

### Comparison With Prior Work

Our findings are largely consistent with related findings from a recent meta-analysis on family therapy, including 3 trials reporting no evidence for a difference at postintervention test between therapist-guided digital versus face-to-face delivery of interventions for youth outcomes and parent outcomes [6]. Similarly, our findings are consistent with findings from a recent meta-analysis on therapist-guided digital parenting interventions that included 4 trials reporting no evidence for a difference at posttest for youth outcomes between therapist-guided digital and face-to-face delivery conditions [7]. None of this prior work, including ours, either strongly supports the superiority of face-to-face over digital delivery conditions or vice versa.

This study adds to research as the first to contribute to the existing body of research from an ST standpoint comparing RCTs of digital and face-to-face delivery modalities of the same intervention. While showing considerable overlap with family therapy, couple therapy, and parenting interventions, ST interventions are characterized by a largely independent theoretical framework and set of therapeutic techniques. Recent reviews in related areas of research [6,7,10,47,73] include interventions with different conceptual backgrounds (eg, family-based cognitive behavioral therapy and the integrated family intervention for child conduct problems [6]) under umbrella terms, such as family therapy or parenting interventions. The theory-driven approach we adopted for this systematic review helps to increase granularity by only including interventions based on ST content and conceptual background.

### Strengths and Limitations

Our systematic review has several strengths. First, we applied rigorous methodology, which was preregistered and followed PRISMA guidelines [26]. Second, we applied a search strategy designed for high sensitivity to 6 databases, with an extensive range of complimentary searches coupled with consistent application of inclusion criteria on study design, intervention type, and delivery modalities, which enabled the descriptive synthesis of published evidence based on diverse target populations, different contexts, and different digital delivery modalities. Third, including ST interventions by identifying CPPs and testing them against our definition criteria ensured high levels of reproducibility and validity.

However, there are also limitations to be considered when interpreting the findings of this study. First, the definition of ST applied here is an adapted version of one of several, at times inconsistent, definitions of ST currently used [74,75]. However, our definition is based on the widely accepted definition by von Sydow et al [19], which has also been used as the basis for large-scale public policy and insurance regulations. Second, the operationalized definition criteria were only applied to identified CPPs of the interventions and for criterion 1 (namely, “perceives behavior and mental symptoms within the context of the social systems people live in”), conceptual background to make inclusion or exclusion judgments during screening. This approach may have excluded additional potentially relevant information about the interventions not provided as part of the description of the intervention itself (eg, the information provided in cited articles). However, we judged this approach to be the most beneficial to this study’s replicability by reducing the arbitrary selection of intervention features as much as possible. Third, given that this study was, to our knowledge, the first to investigate digital versus face-to-face delivery modalities for ST interventions, no a priori categorical constraints were defined with regard to participants, outcomes, or contexts. Instead, constraints were defined post hoc, with the goal of maximizing diversity between and minimizing diversity within subgroups. Of note, despite a few constraints regarding the type of participant population, the sample from which participants were recruited was found to be comparatively narrowly defined (youths or parents of youths). Fourth, while our definition of the minimal important difference is in line with current guidelines [19] and current research in related fields [69], also based on Cohen *d* cutoff of 0.2 for a small effect, there are other approaches, such as anchor-based estimates [76], which might yield different ranges for the minimal important differences for the scales included in this study. However, given the fact that the required data for other approaches toward defining the minimally important differences, such as anchors or expert ratings, are currently not available for all identified outcome measures, our approach based on a Cohen *d* cut-off is acceptable and feasible.

### Generalizability

Some factors limit the generalizability of our findings. First, while the identification of a low number of trials constitutes a finding in its own right, it limits the appropriateness of meta-analytic aggregation. This was exacerbated by the fact that multiple articles with potentially relevant findings reporting on the same trial were identified and that some articles, in some cases, showed inconsistencies in reporting data (eg, on sample size). Second, despite post hoc introduction of categories, diversity within these categories remained substantial (eg, in terms of participants’ race, differences in face-to-face delivery formats, or different outcome domains), further limiting the appropriateness of meta-analytic aggregation. The differential impact of the type of digital modality used in each trial further limits the results of our study, and due to the low number of trials, it was not possible to analyze these tools separately. Third, the high diversity between trials (eg, in terms of delivery modalities, patient features, outcomes, and contexts) combined with the low overall number of trials limits the generalizability of this study’s findings to other populations and delivery contexts. Fourth, the low number of identified trials did not allow for subgroup analyses based on RoB judgments. Fifth, 3 of the 4 trials were conducted in the United States, and 3 of the 4 trials recruited a substantial majority of White participants, further limiting generalizability to other contexts and target populations. Sixth, the influence of factors such as paraverbal communication, subject of discussion, and client-practitioner dynamics is crucial for understanding psychotherapeutic processes. Although RCTs are seen as the gold standard in evidence-based medicine, they may not adequately represent these factors, and the same applies to our study by extension.

### Clinical and Research Implications

This review has some important clinical and research implications. Before digital interventions can be accepted unanimously and unambiguously, solid evidence is needed to be built on specific criteria for evaluating the appropriateness of digital modes of delivering ST interventions. Digital delivery is rapidly increasing despite insufficient evidence on the comparative efficacy of digital and face-to-face delivery modalities of ST. This might explain some of the reported clinicians’ resistance to implementing digital interventions in their practices [77] and should caution against replacing face-to-face with digital delivery modalities without careful consideration. At the same time, given the evidence base supporting the efficacy of digitally delivered systemic psychotherapy (though currently still not as broad as that for face-to-face delivery), the largely inconclusive evidence regarding the comparative efficacy of delivery modalities paired with some indicators for the superiority of digital delivery modalities of ST for certain populations and contexts could also be interpreted as a caution against adopting face-to-face delivery of ST interventions as the clinical default. Our study might serve as a first tentative indicator that different populations benefit differently from different delivery modalities. One could, for instance, look at differences between significant outcomes from the included trials in our study and speculate if parents might be more likely to benefit from face-to-face delivery than youths who, in turn, might be more likely to benefit from digital delivery modalities. Potential avenues for future research in this regard could, for instance, investigate the impact of the parents’ role as facilitators of self-guided group interventions. At this point, these points of departure for future research remain speculative, given the current lack of clear evidence regarding the comparative efficacy of digital and face-to-face delivery modalities of ST. To that end, more studies with equivalence design are required.

According to Greene et al [78], equivalence studies should include 4 key aspects in their design: an active comparator condition, identifying appropriate equivalence margins, calculating adequate sample size estimations, and doing appropriate statistical analysis [78]. Our review highlights the need for research implementing all these key aspects. Furthermore, future research should explore subgroups or conduct sensitivity analyses to understand the limitations and the differential advantages of digital and face-to-face delivery modalities. The questions, such as how different types of digital interventions compare to face-to-face interventions and which intervention is efficacious for target populations, specific contexts, and various modes of digital interventions, including therapist-guided, self-guided, videoconferencing, virtual augmented reality, and smartphone apps, remain unanswered. Moreover, research on digital apps and interventions that, even if not directly involved in therapy, may provide several benefits in supporting those delivering and receiving mental health interventions is required. There is a need to investigate digital modes of delivery that could potentially complement face-to-face interventions through blended forms of care (refer to the study by Erbe et al [79]), specifically in the context of ST. Overall, more high-quality controlled trials using similar outcome measures as well as systematic reviews and meta-analyses are required to scrutinize under what circumstances digital ST interventions work best, especially given the increasing amount of available modes of delivery and the increase in digital delivery of mental health care in general.

### Conclusions

Taken together, our systematic review indicated that the current evidence regarding the comparative efficacy of digital and face-to-face delivery modalities of ST interventions does not allow conclusions to be drawn regarding equivalence or superiority for youth, parent, family functioning, adherence, attrition, or further outcomes. There is a dearth of RCTs comparing the efficacy of comparable ST interventions delivered via both digital and face-to-face delivery modalities. The low number of trials combined with high clinical and methodological diversity between trials did not permit drawing meaningful conclusions from meta-analytic aggregation, and neither equivalence nor superiority of one modality can be excluded at this point. Nonaggregated analyses of MDs and CIs between delivery modalities indicated that neither equivalence nor superiority of either modality can be excluded at this stage for most outcomes, target populations, and contexts. As digital delivery of ST interventions is becoming increasingly ubiquitous, more research is urgently required to investigate how digital delivery of ST interventions compares to face-to-face delivery of the same interventions. There is a need for more research with a particular focus on different types of digital delivery modalities, including blended forms of mental health care, investigating potential variation in efficacy across different settings, populations, outcomes, and contexts.

## Data Availability

The datasets generated and analyzed during this study are available from the corresponding author on reasonable request. A list of articles excluded with respective justifications for exclusions at the full-text screening stage can be made available upon request.

## Appendix

Multimedia Appendix 1 PRISMA (Preferred Reporting Items for Systematic Reviews and Meta-Analyses) 2020 checklist.

Multimedia Appendix 2 Intervention inclusion criterion and constituent primary parts of interventions meeting definition criteria of systemic psychotherapy.

Multimedia Appendix 3 Search terms, structure of search string, and database adaptations.

Multimedia Appendix 4 Data extracted.

Multimedia Appendix 5 Reported statistics on face-to-face versus digital delivery.

Multimedia Appendix 6 Characteristics of included articles.

Multimedia Appendix 7 Outcome measures overview.

Multimedia Appendix 8 Comparison tables for outcomes.

Multimedia Appendix 9 Family functioning outcomes.

Multimedia Appendix 10 Adherence, attrition, and further outcomes.

Multimedia Appendix 11 Screened reviews and meta-analyses.

Multimedia Appendix 12 Flowchart of participant characteristics, measurement time points, intervention delivery type, and superiority or equivalence of delivery modalities. BSI: Brief Symptom Inventory; CES-D: Center for Epidemiological Studies Depression Scale; DD: digital delivery; F2F: face-to-face; F-PST: Family Problem-Solving Therapy; HBI: Health and Behaviour Inventory; P-PE: Parent Rated Program Evaluation; SCL: Symptom Checklist 90-Revised Global Severity Index; SUCCEAT: Supporting Carers of Children and Adolescents with Eating Disorders in Austria; PedsQL: Pediatric Quality of Life Inventory.

## Abbreviations

BFST-D: Behavioral Family Systems Therapy for Diabetes
CPP: constituent primary part
F-PST: Family Problem-Solving Therapy
MD: mean difference
PAAS: Pathways for African American Success
PRISMA: Preferred Reporting Item for Systematic Reviews and Meta-Analyses
RCT: randomized controlled trial
RoB: risk of bias
ST: systemic therapy
SUCCEAT: Supporting Carers of Children and Adolescents with Eating Disorders in Austria
