# Supplementary material for: Comparing Digital Versus Face-to-Face Delivery of Systemic Psychotherapy Interventions: Systematic Review and Meta-Analysis of Randomized Controlled Trials
Source: Interact J Med Res. 2025 Feb 24;14:e46441. doi: 10.2196/46441 (PMC11894358; doi:10.2196/46441)
Supplement: Multimedia Appendix 2 [file ijmr_v14i1e46441_app2.docx]

**Multimedia Appendix 2:** Intervention inclusion criterion and constituent primary parts (CPPs) of interventions meeting definition criteria of systemic therapy

Systemic therapy was operationalized based on 4 adapted definition criteria of systemic therapy (adaptations in square brackets):

We use “systemic/systems-oriented therapy/therapies (ST)” as a general term for a major therapeutic orientation that can be distinguished from other major approaches (e.g., CBT or psychodynamic therapy). We define systemic therapy as a form of psychotherapy that (1) perceives behavior and mental symptoms within the context of the social systems people live in; (2) focuses on interpersonal relations and interactions, social constructions of realities, and[/or] the recursive causality between symptoms and interactions; (3) includes family members and[/or] other important persons (e.g., teachers, friends, professional helpers) directly or indirectly through systemic questioning, hypothesizing, and specific interventions; and (4) appreciates and utilizes clients’ perspectives on problems, resources, and[/or] preferred solutions^1^.

For each intervention, the appropriate unit of primary intervention delivery (as opposed to parts of the intervention labeled “supplementary”, “additional”, or “optional”, etc.) was determined following the study’s authors (e.g., core sessions, core components, etc.). The total number of the intervention’s constituent primary parts (CPP’s) was determined. If the intervention description did not afford determining an appropriate unit of intervention delivery, the total number of CPP’s was defined as 1. At least 2 out of the 4 definition criteria needed to be met by all CPP. Definition criterion (1) could additionally be met by relevant conceptual background provided. At most 2 out of the 4 definition criteria could be met by a minimum of at least half of all CPP’s (or, in case of uneven numbers of CPP’s, the closest possible number below the theoretical half-point). The same CPP could satisfy more than 1 criterion.

**Table 1.** Interventions, identified CPPs against definition criteria

|  |  | Criteria from adapted systemic therapy definition | | | |
| --- | --- | --- | --- | --- | --- |
|  |  |  | | | |
| Intervention | Number and unit of CPPs | (1) Perceives behavior and mental symptoms within the context of the social systems people live in | (2) Focuses on interpersonal relations and interactions, social constructions of realities, and/or the recursive causality between symptoms and interactions | (3) Includes family members and/or other important persons (e.g., teachers, friends, professional helpers) directly or indirectly through systemic questioning, hypothesizing, and specific interventions | (4) Appreciates and utilizes clients’ perspectives on problems, resources, and/or preferred solutions |
|  |  |  |  |  |  |
| BFST-D^2^ | 4 primary intervention components | Family functioning and maladaptive parent-child interactions identified as central barriers to diabetes treatment adherence. | 3 out of 4 CPPs: CPP1: Family problem solving (as a family, defining the problem, generating solutions, making decisions, implementing and monitoring results, and refining ineffective solutions); CPP2: Communication training (instruction, feedback, modeling, rehearsal of approaches towards improving maladaptive communicative patterns); CPP4: Family restructuring (functional and structural approaches towards changing maladaptive or ineffective family system patterns and characteristics such as weak parental coalitions or cross-generational coalitions). | All CPPs are delivered to the caregiver/adolescent dyad. | 2 out of 4 CPPs: CPP1; CPP3: Cognitive restructuring (addressing beliefs, attitudes, and attributions that could negatively affect effective interactions). |
| PAAS^3^ | 6 sessions (with distinct parent, youth, and joint family components) | Adolescent risk behaviours conceptualized within the context of family dynamics as well as larger societal dynamics. Risk behaviour explicitly targeted at individual adolescent, parent, and family levels. | 6 out of 6 CPPs: CPP1: Session 1 (Parent component: “Supportive Parenting”; Family component: Supporting our youth”); CPP2: Session 2 (Parent component: “Establishing Family Rules and Routines Nurturing Involved Parenting”; Youth component: “Self-discovery & Autonomy”; Family component: “Family values”); CPP3: Session 3 (Youth component: “Dealing with unfair situations”; Family component: “Encouraging racial pride”); CPP4: Session 4 (Youth component: “Being cool & smart”; Family component: “Positive, affectionate family relations”); CPP5: Session 5 (Youth component: “Resisting peer pressure”; Family component: “Caregivers and young people working together to protect youth from risk behaviors”); CPP6: Session 6 (Parent component: “Parental protections that reduce high risk behaviors”; Family component: “Our family plan and pledge for positive youth development”). | Parent components of sessions are delivered to more than 1 caregiver per family. Every CPP contained a family component in which intervention was delivered to caregivers and adolescents jointly. | 4 out of 6 CPPs: CPP2: Session 2 (Parent component; Youth component; Family component); CPP3: Session 3 (Parent component: “Adaptive Racial Socialization and Encouraging Racial Pride”, Youth component; Family component); CPP4: Session 4 (Youth component: “Being cool & smart”; Family component: “Positive, affectionate family relations”); CPP6: Session 6 (Youth component: “Dealing with sexual and substance and drug use temptations”; Family component) |
| F-PST^4^ | 10 core sessions | Caregiver distress and maladaptive caregiver-adolescent interactions conceptualized as central factors in adolescent’s recovery from traumatic brain injury (TBI). Intervention designed to facilitate recovery by targeting family and caregiver functioning. | 9 out of 10 CPP: CPP2: Session 2 (“Problem Solving” as family; family problem solving technique used as part of every subsequent CPP); CPP4: Session 4 (“Working with the School after TBI”); CPP 6: Session 6 (“Controlling Your Anger & Improving Communication”); CPP7: Session 7 (“Listening, Talking, Reading Non-Verbal Cues”); CPP8: Session 8 (“Social Behavior and Joining a Group”) | Intervention delivered to adolescents with TBI and their families conjointly. | 9 out of 10 CPP: CPP2 and all subsequent CPPs. |
| SUCCEAT^5^ | 8 sessions | Psychological burden on caregivers of patients with AN conceptualized within the context of interactional dynamics between caregiver and AN patient. Intervention techniques used to alleviate caregivers’ psychological burden target interactions with AN patient. | 5 out 8 CPP: CPP1: Session 1 („Interpersonal Factors“); CPP3: Session 3 (“Stages of Change”); CPP4: Session 4 (“Motivational Interviewing I”); CPP5: Session 5 (“Motivational Interviewing II”); CPP6: Session 6 (“Stress and Emotions”) | Interventions delivered to caregivers alone, but all CPP involve AN patient indirectly. | 5 out of 8 CPPs: CPP1; CPP2: Session 2 (“Brain and Compassion”); CPP4; CPP5; CPP6 |

# References

1. von Sydow K, Retzlaff R, Beher S, Haun MW, Schweitzer J. The efficacy of systemic therapy for childhood and adolescent externalizing disorders: a systematic review of 47 RCT. *Fam Process*. Dec 2013;52(4):576-618. doi:10.1111/famp.12047

2. Duke DC, Wagner DV, Ulrich J, Freeman KA, Harris MA. Videoconferencing for Teens With Diabetes: Family Matters. *J Diabetes Sci Technol*. Jul 2016;10(4):816-23. doi:10.1177/1932296816642577

3. Murry VM, Berkel C, Liu N. The Closing Digital Divide: Delivery Modality and Family Attendance in the Pathways for African American Success (PAAS) Program. *Prev Sci*. Jul 2018;19(5):642-651. doi:10.1007/s11121-018-0863-z

4. Kurowski BG ST, Taylor HG, et al. Comparative effectiveness of family problem-solving therapy (F-PST) for adolescents after traumatic brain injury: Protocol for a randomized, multicenter, clinical trial. *Contemp Clin Trials Commun*. 2018;10:111-120. doi:10.1016/j.conctc.2018.04.001

5. Franta C PJ, Waldherr K, et al. . Supporting Carers of Children and Adolescents with Eating Disorders in Austria (SUCCEAT): Study protocol for a randomised controlled trial. . *Eur Eat Disord Rev*. 2018;26(5):447-461. doi:10.1002/erv.2600
