# Supplementary material for: Comparing Digital Versus Face-to-Face Delivery of Systemic Psychotherapy Interventions: Systematic Review and Meta-Analysis of Randomized Controlled Trials
Source: Interact J Med Res. 2025 Feb 24;14:e46441. doi: 10.2196/46441 (PMC11894358; doi:10.2196/46441)
Supplement: Multimedia Appendix 3 [file ijmr_v14i1e46441_app3.docx]

**Multimedia Appendix 3:** Search terms, structure of search string, and database adaptations

**Table 1.** Search terms and structure of search string

| # | Search term^a^ | Search target | Reference/function |
| --- | --- | --- | --- |
|  |  |  |  |
| 1 | family | Title and abstract | von Sydow et al., 2013^1^ |
| 2 | couple | Title and abstract | von Sydow et al., 2013^1^ |
| 3 | parent | Title and abstract | Retzlaff, 2013^2^ |
| 4 | guardian | Title and abstract | von Sydow et al., 2013^1^ |
| 5 | systemic | Title and abstract | von Sydow et al., 2013^1^ |
| 6 | structural | Title and abstract | von Sydow et al., 2013^1^ |
| 7 | strategic | Title and abstract | von Sydow et al., 2013^1^ |
| 8 | solution-focused | Title and abstract | von Sydow et al., 2013^1^ |
| 9 | multisystemic | Title and abstract | Hefti S, Pérez T, Fürstenau U, Rhiner B, Swenson CC, Schmid M., 2020^3^ |
| 10 | McMaster model | Title and abstract | von Sydow et al., 2013^1^ |
| 11 | narrative therapy | Title and abstract and MeSH | von Sydow et al., 2013^1^ |
| 12 | interpersonal therapy | Title and abstract^b^ | Tomm et al., 2014^4^ |
| 13 | paradoxical intervention | Title and abstract | Ataoglu et al., 2003^5^ |
| 14 | conjoint | Title and abstract | Pinsof et al., 2018^6^ |
| 15 | interaction therapy | Title and abstract | Hale & Frusha, 2016^7^ |
| 16 | Problem-solving therapy | Title and abstract | Vitry et al., 2021^8^ |
| 17 | psychotherapy | MeSH | Increase specificity by defining intervention domain of interest |
| 18 | stress, psychological | MeSH | Increase specificity by defining intervention domain of interest |
| 19 | psychosomatic medicine | MeSH | Increase specificity by defining intervention domain of interest |
| 20 | psychotherapy | Title and abstract | Increase specificity by defining intervention domain of interest |
| 21 | #17 OR #18 OR #19 # 20 |  | #17-20 compiled as specificity term |
| 22 | #1 OR #2 OR #3 OR #4 OR #5 OR #6 OR #7 OR #8 OR #9 OR #10 OR #11 OR #12 OR #13 OR #14 OR #15 OR #16 |  | #1-16 compiled as intervention term |
| 23 | #21 AND #22 |  | Intervention term and specificity term compiled |
| 24 | computer | Title and abstract | Smoktunowicz 2020^9^ |
| 25 | tele | Title and abstract | Smoktunowicz 2020^9^ |
| 26 | cyber | Title and abstract | Smoktunowicz 2020^9^ |
| 27 | digital | Title and abstract | Smoktunowicz 2020^9^ |
| 28 | online | Title and abstract | Smoktunowicz 2020^9^ |
| 29 | web | Title and abstract | Smoktunowicz 2020^9^; Baumel et al., 2016^10^ |
| 30 | smartphone | Title and abstract | Baumel et al., 2016^10^ |
| 31 | internet | Title and abstract | Smoktunowicz 2020^9^ |
| 32 | virtual | Title and abstract | Smoktunowicz 2020^9^ |
| 33 | remote | Title and abstract | Smoktunowicz 2020^9^ |
| 34 | distance | Title and abstract | Smoktunowicz 2020^9^ |
| 35 | videoconference | Title and abstract | Smoktunowicz 2020^9^ |
| 36 | avatar | Title and abstract | Smoktunowicz 2020^9^ |
| 37 | e-mail | Title and abstract | Smoktunowicz 2020^9^ |
| 38 | chat | Title and abstract | Knopp et al., 2021^11^; Hoermann et al., 2017^12^ |
| 39 | e-health | Title and abstract | Baumel et al., 2016^10^ |
| 40 | e-aid | Title and abstract | Smoktunowicz 2020^9^ |
| 41 | e-therapy | Title and abstract | Smoktunowicz 2020^9^ |
| 42 | e-mental heath | Title and abstract | Baumel et al., 2016^10^ |
| 43 | e-counseling | Title and abstract | Smoktunowicz 2020^9^ |
| 44 | mHealth | Title and abstract | Baumel et al., 2016^10^ |
| 45 | interapy | Title and abstract | Smoktunowicz 2020^9^ |
| 46 | medicine 2.0 | Title and abstract | Smoktunowicz 2020^9^ |
| 47 | minimal contact intervention | Title and abstract | Smoktunowicz 2020^9^ |
| 48 | #24 OR #25 OR #26 OR #27 OR #28 OR #29 OR #30 OR #31 OR #32 OR #33 OR #34 OR #35 OR #36 OR #37 OR #38 OR #39 OR #40 OR #41 OR #42 OR #43 OR #44 OR #45 OR #46 OR #47 |  | #24-47 compiled as delivery modality term |
| 49 | #23 AND #48 |  | Intervention, specificity, and delivery modality terms compiled |

^a^: Permutations (e.g., separate terms including “therapy” and “psychotherapy” or truncation) added where appropriate (see search strings below).

^b^: The intended target for this search term was title and abstract

**PubMed**

((family[tiab] OR couple*[tiab] OR parent*[tiab] OR guardian*[tiab] OR systemic*[tiab] OR structural*[tiab] OR strategic*[tiab] OR "solution focused"[tiab] OR multisystemic[tiab] OR "McMaster model"[tiab] OR "narrative therapy"[mesh] OR "narrative therapy”[tiab] OR "narrative psychotherapy"[tiab] OR "interpersonal psychotherapy"[mesh] OR "interpersonal therapy"[tiab] OR "interpersonal relations therapy"[tiab] OR "interpersonal psychotherapy" OR "paradoxical intervention"[tiab] OR conjoint[tiab] OR "interaction therapy"[tiab] OR "interaction psychotherapy"[tiab] OR "problem-solving therapy"[tiab] OR PST[tiab] OR FPST[tiab] OR "F-PST"[tiab] OR "problem-solving psychotherapy"[tiab] OR "problem-solving skills"[tiab]) AND (psychotherapy[mesh] OR "stress, psychological"[mesh] OR "psychosomatic medicine"[mesh] OR psychotherap*[tiab])) AND ((computer*[tiab] OR tele*[tiab] OR cyber*[tiab] OR digital*[tiab] OR online*[tiab] OR web[tiab] OR web-based[tiab] OR website*[tiab] OR smartphone*[tiab] OR internet*[tiab] OR virtual*[tiab] OR remote*[tiab] OR distan*[tiab] OR videoconferenc*[tiab] OR avatar*[tiab] OR e-mail*[tiab] OR chat*[tiab] OR "e-health"[tiab] OR "e-aid"[tiab] OR "e-therapy"[tiab] OR "e-mental heath"[tiab] OR "e-counseling"[tiab] OR "e-counselling"[tiab] OR mHealth[tiab] OR "m-health"[tiab] OR interapy[tiab] OR "medicine 2.0"[tiab] OR "minimal contact intervention"[tiab]))

**Cochrane Library (via WILEY)**

| Search  number | Search item |
| --- | --- |
| #1 | ((family OR couple* OR parent* OR guardian* OR systemic* OR structural* OR strategic* OR “solution?focused” OR multisystemic OR “McMaster model” OR narrative OR interpersonal OR paradoxical OR conjoint OR interaction) NEAR/4 (psychotherap* OR therap* OR intervention* OR treatment* OR problem?solving*)):ti,ab |
| #2 | MeSH descriptor: [Narrative Therapy] explode all trees |
| #3 | (“PST” OR “FPST” OR “F-PST”):ti,ab |
| #4 | #1 OR #2 OR #3 |
| #5 | MeSH descriptor: [Psychotherapy] explode all trees |
| #6 | MeSH descriptor: [Stress, Psychological] explode all trees |
| #7 | MeSH descriptor: [Psychosomatic Medicine] explode all trees |
| #8 | psychotherap*:ti,ab |
| #9 | #4 AND (#5 OR #6 OR #7 OR #8) |
| #10 | #9 AND (computer* OR tele* OR cyber* OR digital* OR online* OR web OR “web-based” OR website* OR smartphone* OR internet* OR virtual* OR remote* OR distan* OR videoconferenc* OR avatar* OR e-mail* OR chat* OR “e-health” OR “e-aid” OR “e-therapy” OR “e-mental heath” OR “e-counsel?ing” OR “m?Health” OR interapy OR “medicine 2.0” OR “minimal contact intervention”):ti,ab |

**PsycInfo, PSYNDEX, and CINAHL (via EBSCOhost)**

Includes dissertations and theses (Dissertation Abstracts International: Section B: The Sciences and Engineering)

((ti ((family OR couple* OR parent* OR guardian* OR systemic* OR structural* OR strategic* OR “solution#focused” OR multisystemic OR “McMaster model“ OR narrative OR interpersonal OR paradoxical OR conjoint OR interaction) N4 (psychotherap* OR therap* OR intervention* OR treatment* OR problem#solving*)) OR ab ((family OR couple* OR parent* OR guardian* OR systemic* OR structural* OR strategic* OR “solution#focused” OR multisystemic OR “McMaster model“ OR narrative OR interpersonal OR paradoxical OR conjoint OR interaction) N4 (psychotherap* OR therap* OR intervention* OR treatment* OR problem#solving*))) AND (ti (computer* OR tele* OR cyber* OR digital* OR online* OR web OR web-based OR website* OR smartphone* OR internet* OR virtual* OR remote* OR distan* OR videoconferenc* OR avatar* OR e-mail* OR chat* OR “e-health” OR “e-aid” OR “e-therapy” OR “e-mental health” OR “e-counsel#ing” OR “m#Health” OR interapy OR “medicine 2.0” OR “minimal contact intervention”) OR ab (computer* OR tele* OR cyber* OR digital* OR online* OR web OR web-based OR website* OR smartphone* OR internet* OR virtual* OR remote* OR distan* OR videoconferenc* OR avatar* OR e-mail* OR chat* OR “e-health” OR “e-aid” OR “e-therapy” OR “e-mental health” OR “e-counsel#ing” OR “m#Health” OR interapy OR “medicine 2.0” OR “minimal contact intervention”)))

**Embase (via OVID)**

(((((family OR couple* OR parent* OR guardian* OR systemic* OR structural* OR strategic* OR solution?focused OR multisystemic OR McMaster model OR narrative OR interpersonal OR paradoxical OR conjoint OR interaction) ADJ4 (psychotherap* OR therap* OR intervention* OR treatment* OR problem?solving*)) OR PST OR F?PST).ti,ab. OR exp narrative therapy/) AND (exp psychotherapy/ OR exp stress, psychological/ OR exp Psychosomatic Medicine/ OR (psychotherap*).ti,ab.) AND (computer* OR tele* OR cyber* OR digital* OR online* OR web OR web-based OR website* OR smartphone* OR internet* OR virtual* OR remote* OR distan* OR videoconferenc* OR avatar* OR e-mail* OR chat* OR e-health OR e-aid OR e-therapy OR e-mental health OR e-counsel?ing OR m?Health OR interapy OR minimal contact intervention).ti,ab.)

# **References**

1. von Sydow K, Retzlaff R, Beher S, Haun MW, Schweitzer J. The efficacy of systemic therapy for childhood and adolescent externalizing disorders: a systematic review of 47 RCT. *Fam Process*. Dec 2013;52(4):576-618. doi:10.1111/famp.12047

2. Retzlaff R, von Sydow K, Beher S, Haun MW, Schweitzer J. The efficacy of systemic therapy for internalizing and other disorders of childhood and adolescence: a systematic review of 38 randomized trials. *Fam Process*. Dec 2013;52(4):619-52. doi:10.1111/famp.12041

3. Hefti S PT, Fürstenau U, Rhiner B, Swenson CC, Schmid M. Multisystemic Therapy for Child Abuse and Neglect: Do Parents Show Improvement in Parental Mental Health Problems and Parental Stress? *J Marital Fam Ther*. 2020;46(1):95-109. doi:10.1111/jmft.12367

4. Tomm K. Introducing the IPscope: A systemic assessment tool for distinguishing

interpersonal patterns. In: In K. M. Tomm SSG, D. Wulff, & T. Strong, ed. *Patterns*

*in interpersonal interactions: Inviting relational understandings for therapeutic change*. Routledge; 2014:13-35.

5. Ataoglu A OA, Icmeli C, Ozbulut O. Paradoxical therapy in conversion reaction. *J Korean Med Sci*. 2003;18(4):581-584. doi:10.3346/jkms.2003.18.4.581

6. Pinsof W, Breunlin D, Russell W, Lebow J, Chambers A. *Integrative systemic therapy: Metaframeworks for problem solving with individuals, couples and families*. American Psychological Association.; 2018.

7. Hale D, Frusha C. MRI Brief Therapy: A Tried and True Systemic Approach. *Journal of Systemic Therapies*. 2016;35:14-24. doi:10.1521/JSYT.2016.35.2.14

8. Vitry G, Pakrosnis R, Brosseau O, Duriez N. Effectiveness and Efficiency of Strategic and Systemic Therapy in Naturalistic Settings: Preliminary Results from a Systemic Practice Research Network (SYPRENE). *Journal of Family Therapy*. 2021;0:1-2. doi: 10.1111/1467-6427.12343

9. Smoktunowicz E, Barak A, Andersson G. Consensus statement on the problem of terminology in psychological interventions using the internet or digital components. *Internet Interv*. 2020;21:100331. doi:10.1016/j.invent.2020.100331

10. Baumel A, Correll CU, Birnbaum M. Adaptation of a peer based online emotional support program as an adjunct to treatment for people with schizophrenia-spectrum disorders. *Internet Interv*. May 2016;4:35-42. doi:10.1016/j.invent.2016.03.003

11. Knopp K SJ, Khalifian C, Grubbs K, Morland LA, Depp C. . Digital interventions for couples: State of the field and future directions. *Couple and Family Psychology: Research and Practice*. 2021;doi:10.1037/cfp0000213

12. Hoermann S MK, Milne DN, Calvo RA. Application of synchronous text-based dialogue systems in mental health interventions: systematic review. *J Med Internet Res* 2017;19(8):e267. doi:10.2196/jmir.7023
