# Supplementary material for: Comparing Digital Versus Face-to-Face Delivery of Systemic Psychotherapy Interventions: Systematic Review and Meta-Analysis of Randomized Controlled Trials
Source: Interact J Med Res. 2025 Feb 24;14:e46441. doi: 10.2196/46441 (PMC11894358; doi:10.2196/46441)
Supplement: Multimedia Appendix 4 [file ijmr_v14i1e46441_app4.docx]

**Multimedia Appendix 4:** Screened reviews and meta-analyses

**Table 1.** Screened articles and number of publications identified

| Number of publications identified | Article Reference |
| --- | --- |
|  |  |
|  |  |
| 0 | ^1^von Sydow K, Retzlaff R, Beher S, Haun MW, Schweitzer J. The efficacy of systemic therapy for childhood and adolescent externalizing disorders: a systematic review of 47 RCT. *Fam Process*. 2013;52(4):576-618. doi:10.1111/famp.12047 |
| 0 | ^2^Baumel A, Pawar A, Kane JM, Correll CU. Digital Parent Training for Children with Disruptive Behaviors: Systematic Review and Meta-Analysis of Randomized Trials. *J Child Adolesc Psychopharmacol*. 2016;26(8):740-749. doi:10.1089/cap.2016.0048 |
| 0 | ^3^Leijten P, Gardner F, Melendez-Torres GJ, et al. Meta-Analyses: Key Parenting Program Components for Disruptive Child Behavior. *J Am Acad Child Adolesc Psychiatry*. 2019;58(2):180-190. doi:10.1016/j.jaac.2018.07.900 |
| 0 | ^4^de Boer K, Muir SD, Silva SSM, et al. Videoconferencing psychotherapy for couples and families: A systematic review. *J Marital Fam Ther*. 2021;47(2):259-288. doi:10.1111/jmft.12518 |
| 0 | ^5^Helps S, Grinney MLC. Synchronous Digital Couple and Family Psychotherapy: A Meta-narrative Review. Journal of Family Therapy. 2021;43: 185-214. doi: 10.1111/1467-6427.12333 |
| 0 | ^6^Carr A. Couple Therapy, Family Therapy and Systemic Interventions for Adult-focused Problems: The Current Evidence Base. Journal of Family Therapy. 2018;40: 492-536. doi: 10.1111/1467-6427.12225 |
| 0 | ^7^Carr A. Family Therapy and Systemic Interventions for Child-focused Problems: The Current Evidence Base. Journal of Family Therapy. 2019;41: 153-213. doi: 10.1111/1467-6427.12226 |
| 0 | ^8^Gelin Z, Cook-Darzens S, Simon Y, Hendrick S. Two models of multiple family therapy in the treatment of adolescent anorexia nervosa: a systematic review. *Eat Weight Disord*. 2016;21(1):19-30. doi:10.1007/s40519-015-0207-y |
| 0 | ^9^Pinquart M, Oslejsek B, Teubert D. Efficacy of systemic therapy on adults with mental disorders: A meta-analysis. *Psychother Res*. 2016;26(2):241-257. doi:10.1080/10503307.2014.935830 |
| 0 | ^10^Retzlaff R, von Sydow K, Beher S, Haun MW, Schweitzer J. The efficacy of systemic therapy for internalizing and other disorders of childhood and adolescence: a systematic review of 38 randomized trials. *Fam Process*. 2013;52(4):619-652. doi:10.1111/famp.12041 |
| 0 | ^11^Sourander A, McGrath PJ, Ristkari T, et al. Internet-Assisted Parent Training Intervention for Disruptive Behavior in 4-Year-Old Children: A Randomized Clinical Trial. *JAMA Psychiatry*. 2016;73(4):378-387. doi:10.1001/jamapsychiatry.2015.3411 |
| 0 | ^12^Köhnen M, Kriston L, Härter M, Dirmaier J, Liebherz S. Rationale and design of a systematic review: effectiveness and acceptance of technology-based psychological interventions in different clinical phases of depression management. *BMJ Open*. 2019;9(3):e028042. Published 2019 Mar 27. doi:10.1136/bmjopen-2018-028042 |

**References**

1. von Sydow K, Retzlaff R, Beher S, Haun MW, Schweitzer J. The efficacy of systemic therapy for childhood and adolescent externalizing disorders: a systematic review of 47 RCT. *Fam Process*. 2013;52(4):576-618. doi:10.1111/famp.12047
2. Baumel A, Pawar A, Kane JM, Correll CU. Digital Parent Training for Children with Disruptive Behaviors: Systematic Review and Meta-Analysis of Randomized Trials. *J Child Adolesc Psychopharmacol*. 2016;26(8):740-749. doi:10.1089/cap.2016.0048
3. Leijten P, Gardner F, Melendez-Torres GJ, et al. Meta-Analyses: Key Parenting Program Components for Disruptive Child Behavior. *J Am Acad Child Adolesc Psychiatry*. 2019;58(2):180-190. doi:10.1016/j.jaac.2018.07.900
4. de Boer K, Muir SD, Silva SSM, et al. Videoconferencing psychotherapy for couples and families: A systematic review. *J Marital Fam Ther*. 2021;47(2):259-288. doi:10.1111/jmft.12518
5. Helps S, Grinney MLC. Synchronous Digital Couple and Family Psychotherapy: A Meta-narrative Review. Journal of Family Therapy. 2021;43: 185-214. doi: 10.1111/1467-6427.12333
6. Carr A. Couple Therapy, Family Therapy and Systemic Interventions for Adult-focused Problems: The Current Evidence Base. Journal of Family Therapy. 2018;40: 492-536. doi: 10.1111/1467-6427.12225
7. Carr A. Family Therapy and Systemic Interventions for Child-focused Problems: The Current Evidence Base. Journal of Family Therapy. 2019;41: 153-213. doi: 10.1111/1467-6427.12226
8. Gelin Z, Cook-Darzens S, Simon Y, Hendrick S. Two models of multiple family therapy in the treatment of adolescent anorexia nervosa: a systematic review. *Eat Weight Disord*. 2016;21(1):19-30. doi:10.1007/s40519-015-0207-y
9. Pinquart M, Oslejsek B, Teubert D. Efficacy of systemic therapy on adults with mental disorders: A meta-analysis. *Psychother Res*. 2016;26(2):241-257. doi:10.1080/10503307.2014.935830
10. Retzlaff R, von Sydow K, Beher S, Haun MW, Schweitzer J. The efficacy of systemic therapy for internalizing and other disorders of childhood and adolescence: a systematic review of 38 randomized trials. *Fam Process*. 2013;52(4):619-652. doi:10.1111/famp.12041
11. Sourander A, McGrath PJ, Ristkari T, et al. Internet-Assisted Parent Training Intervention for Disruptive Behavior in 4-Year-Old Children: A Randomized Clinical Trial. *JAMA Psychiatry*. 2016;73(4):378-387. doi:10.1001/jamapsychiatry.2015.3411
12. Köhnen M, Kriston L, Härter M, Dirmaier J, Liebherz S. Rationale and design of a systematic review: effectiveness and acceptance of technology-based psychological interventions in different clinical phases of depression management. *BMJ Open*. 2019;9(3):e028042. Published 2019 Mar 27. doi:10.1136/bmjopen-2018-028042
