# Supplementary material for: Comparing Digital Versus Face-to-Face Delivery of Systemic Psychotherapy Interventions: Systematic Review and Meta-Analysis of Randomized Controlled Trials
Source: Interact J Med Res. 2025 Feb 24;14:e46441. doi: 10.2196/46441 (PMC11894358; doi:10.2196/46441)
Supplement: Multimedia Appendix 5 [file ijmr_v14i1e46441_app5.docx]

**Multimedia Appendix 5:** Data Extracted

| **Trial/study cluster** | **Salient characteristics/clinical symptoms of index patients** | **Intervention** | **Number and duration of sessions** | **Individual or group receiving treatment** | **Face-to-face implementation** | **Technological implementation** | **Article** | **N** | **Index patient age (mean years)** | **Index patient sex** | **Index patient ethnicity** | **Attrition** | **Measure(s)** | **Time points examined** | **Statistical analyses** | **Face-to-face vs. digital comparison** | **Results** | **Key conclusions** |
| --- | --- | --- | --- | --- | --- | --- | --- | --- | --- | --- | --- | --- | --- | --- | --- | --- | --- | --- |
|  |  |  |  |  |  |  | Duke et al. (2016)^1^  Harris, Freeman, and Duke (2015)^2^ | 90 (46 digital delivery condition; 44 face-to-face delivery condition)  90 (46 digital delivery condition; 44 face-to-face delivery condition) | Digital delivery condition: 14.94 (SD = 1.77); face-to-face delivery condition: 15.04 (SD = 1.79)  Digital delivery condition: 14.94 (SD = 1.77); face-to-face delivery condition: 15.04 (SD = 1.79) | Digital delivery condition: 17 female, 29 male; face-to-face delivery condition: 18 female, 26 male. No stat. sig. difference between conditions |  | Digital delivery condition: 17; face- to-face delivery condition: 9 (both from baseline to follow-up). Stat. sig. difference between conditions (CHI-SQUARE(1) = 10.88; P = 0.001) | Y-HHI, Y-CBQ, Y-AIS, P-HHI, P-CBQ, P-AIS  Y-DSMP, P-DSMP,  HbA1c | Pre, post (4 weeks), follow-up (12 weeks)  Pre, post (4 weeks), follow-up (12 weeks) | ITT analyses with pooled means to impute missing data; between groups repeated measures ANOVA  ITT analyses with pooled means to impute missing data; between groups repeated measures ANOVA | Y-HHI: F(1) = 0.08, P = .78; Y-CBQF:  (1) = 0.32, P = .57; Y-AIS: F(1) = 3.87,  P = .052; P-HHI: F(1) = 0.98, P = .33;  P-CBQ: F(1) = 0.27, P = .60; P-AIS: F  (1) = 1.00, P = .32 | No stat. sig. differences between conditions | Effectiveness of face-to-face BFST-D can be retained in digital delivery modality  BFST-D effective across face-to- face and digital delivery modalities, both delivery modalities combined achieved small, but significant improvements |
|  |  |  |  |  |  |  |  |  |  |  |  |  |  |  |  | "no between-group differences in treatment effects for adherence and glycemic control (F(1) = 0.09; P =  0.77)" (p.1430) | No stat. sig. differences between conditions |  |
| BFST-D | Type 1 diabetes with suboptimal glycemic control (HbA1c ≥ 9.0%) | Behavioral family systems therapy– diabetes | Digital delivery condition: 5.84 (SD = 3.25), face-to-face delivery condition: 6.82 (SD = 3.39), not stat. sig. different; maximum number of sessions: 10; duration: 1-1.5 hours per session | Adolescents and at least one primary caregiver they resided with | Clinic-based visits | Online video conferencing software developed for personal and business applications | Freeman, Duke, and Harris (2013)^3^ | 92 randomized  (47 digital delivery condition; 45 face-to-face delivery condition), 72 included in analysis | Digital delivery condition: 14.9 (SD = 1.9); face-to-face delivery condition: 15.2 (SD = 1.8). No stat. sig. difference (t = -0.034) | Digital delivery condition: 13 female, 19 male; face-to-face delivery condition: 16 female, 23 male. Stat. sig. difference between groups (CHI-SQUARE P = 0.001) |  | Digital delivery condition: 15; face- to-face delivery condition: 6 (both from baseline to post) | Y-WAI, P-WAI | After session 5, post- treatment (4 weeks); only post-treatment used for analysis | Independent samples t-tests | Y-WAI (all subscales): t = 0.834, P- WAI (all subscales): t = 0.528 | No stat. sig. differences between conditions | No significant differences in WAI scores across delivery modalities. For both delivery modalities combined and controlling for baseline HbA1c, number of sessions completed and Y- WAI "goal" subscale (r = 0.443, p = .005)  and Y-WAI total score (r = 0.322, p  = 0.48) were significantly  correlated. |
|  |  |  |  |  |  |  | Riley et al. (2015)^4^ | 90 randomized,  82 included across delivery conditions (only those included with at least 1 obtained CDI measure) | 14.1 across conditions (participants 18 years or older excluded) | 34 female, 48 male across conditions |  | In addition to attrition reported in other articles in BFST-D category: 5 excluded at baseline (at least 18 years, too old for CDI), 3 dropped out before completing CDI baseline measure | CDI | Pre, post (4 weeks), follow-up (12 weeks) | No information provided | "Subsequent between-group analyses have found no differences between groups on measures of depressive symptoms and family processes." (p. 1436) | No stat. sig. differences between conditions | Primary focus of study on relationship between CDI scores and family functioning scores for both delivery modalities combined. No differences found between delivery modalities. |
| PAAS | Rural African American Youth | Pathways to African American Success | 6 sessions (with concurrent separate parts for adolescents and caregivers and an additional conjoint part for everyone); duration per session digital delivery condition: 1.5 hours, duration per session face-to-face delivery condition: 2 hours; see Murry et al. (2018) for more results | At least one caregiver and the adolescent, siblings if available | Role-playing activities, guided discussions, and facilitators responding to questions, delivered to groups of roughly 12 families | Families received individual password to virtual environment in which a "highway to success" maps out session topics, visually representing connections between decisions and their consequences. The environment includes virtual characters which resemble members of the youth's community. Participants have options to customize the appearance of their avatar. They can use this avatar to interact with the virtual characters via  pre-specified responses. Family discussion activities are presented as questions for 3 minutes each on the screen, giving the family time to work on those during that time. Model discussions between virtual characters are provided upon request. | Murry et al. (2019a)^5^ | 421 randomized  (141 digital delivery condition; 141 face-to-face condition; 136 resource control) | "6th-grade" | Digital delivery condition: 53% female [and 47% male?]; face-to- face delivery condition: 55% female [and 45% male?] |  |  | Primary outcomes: Carver Caregiver Support scale, Discussion Quality Scale (and subscales: FoC, DQ, CiC),  expanded version of Substance Use Rules Communication Scale from Strengthening Families, Frequency of Sexual Communication scale, celebration of racial heritage subscale of Racial Socialization Scale, Substance Intention Questions scale, Affiliation with Deviant Peers scale.  Secondary outcomes: Sexual Risk Survey, Monitoring the Future scale | Pre, post (M = 14.5 (SD  = 4.4) months), follow-up (22.6 (SD = 3.7)  months). Pre to post for primary parent and youth outcomes, pre to follow- up for secondary outcomes | Full Information Maximum Likelihood imputation of missing data; structural equation modelling | Primary outcomes: Significant improvement on latent construct general supportive parenting for face- to-face delivery condition (i.e., caregiver support, open communication, frequency of communication): beta = .12; 95% CI =  .02, .20; p = .02. Significant improvements on latent construct parenting with respect to sensitive topics for digital delivery condition: beta = .30; 95% CI = .09, .54; p = .03.  Significant decline in behavioral intent to engage in risk behaviors for digital delivery condition: beta = .12; 95% CI  =.20, .01; p = .04. Significant increase in affiliation with deviant peers in face- to-face delivery condition: beta = .16; 95% CI = .06, .27; p = .002.  Secondary outcomes: Significant decrease in risk behavior for digital delivery modality: beta = .17; 95% CI =  .31, .04; p = .04. Not significant for face-to-face delivery condition (beta =  .05; 95% CI = .20, .11; p = .58). | Primary outcomes: Significant improvement on latent construct general supportive parenting for face- to-face delivery condition (i.e., caregiver support, open communication, frequency of communication). Significant improvements on latent construct parenting with respect to sensitive topics for digital delivery condition.  Significant decline in behavioral intent to engage in risk behaviors for digital delivery condition. Significant increase in affiliation with deviant peers in face- to-face delivery condition. Secondary outcomes: Significant decrease in risk behavior for digital delivery modality. Not significant for face-to-face delivery condition. | Compared to resource control, digital delivery was effective on parenting and adolescent outcomes. Some differential effects between Digital and face-to-face delivery modalities were found |
|  |  |  |  |  |  |  | Murry et al. (2019b)^6^ | 412 randomized  (138 digital delivery condition; 137 face-to-face delivery condition; 137 resource comparison) | 11 across conditions | 54% females across conditions |  | Digital delivery condition: 14; face- to-face delivery condition: 36; resource comparison: 28 (from baseline to post) | Y-AN/EaRE, Y-OSFC, Y-FoC, Y-DQ, Y-CiC, Y-REI, P-AN/EaRE, P- OSFC, P-FoC, P-DQ, P-CiC | Pre, post (6 months) | Full Information Maximum Likelihood imputation of missing data; standardized mean differences, reported as unadjusted effect sizes with confidence intervals | Y-REI was significantly lower in the digital delivery modality condition compared to the face-to-face delivery modality condition (d = -y.36, 95% CI [y-.63, y-.10], p < .05). No other stat. sig. differences at p < .05 level. | Youth randomized to the digital delivery condition reported stat. sig. lower levels of Risk Engagement Intentions than those in the face-to- face delivery condition. No other significant differences between conditions. | Both delivery modalities of the PAAS programme were effective in facilitating aspects of constructive conversations in families and facilitating the internalization of norms, expectations and values. The programme led to a stat. sig. reduction in youth's intentions to engage in risky behaviors only in the digital delivery condition. |
|  |  |  |  |  |  |  | Murry et al. (2018)^7^ |  | M = 11.4 | -- |  |  | Attendance (enrollment, number of sessions attended, retention at least 50% of sessions, retention across all sessions, drop out after 1 session) | n/a | Simple linear regression | Parents assigned to the digital delivery condition were more likely to enroll (71% vs. 57%). They attended a higher number of sessions [M = 4 (SD  = 3) vs. M = 2 (SD = 2). They were more likely to be retained for at least half of the sessions (67% vs. 45%) and retained for all sessions (52% vs. 16%). They were less likely to drop out  after one session (1% vs. 7%). | Parents assigned to the digital delivery condition were more likely to enroll.  They attended a higher number of sessions. They were more likely to be retained for at least half of the sessions and retained for all sessions. They were less likely to drop out after one session. |  |

**References**

1. Duke DC, Wagner DV, Ulrich J, Freeman KA, Harris MA. Videoconferencing for Teens With Diabetes: Family Matters. *J Diabetes Sci Technol*. Jul 2016;10(4):816-23. doi:10.1177/1932296816642577
2. Harris MA, Freeman KA, Duke DC. Seeing Is Believing: Using Skype to Improve Diabetes Outcomes in Youth. *Diabetes Care*. Aug 2015;38(8):1427-34. doi:10.2337/dc14-2469
3. Freeman KA, Duke DC, Harris MA. Behavioral health care for adolescents with poorly controlled diabetes via Skype: does working alliance remain intact? *J Diabetes Sci Technol*. May 1 2013;7(3):727-35. doi:10.1177/193229681300700318
4. Riley AR, Duke DC, Freeman KA, Hood KK, Harris MA. Depressive Symptoms in a Trial Behavioral Family Systems Therapy for Diabetes: A Post Hoc Analysis of Change. *Diabetes Care*. Aug 2015;38(8):1435-40. doi:10.2337/dc14-2519
5. Murry VM, Berkel C, Inniss-Thompson MN, Debreaux ML. Pathways for African American Success: Results of Three-Arm Randomized Trial to Test the Effects of Technology-Based Delivery for Rural African American Families. *J Pediatr Psychol*. Apr 1 2019a;44(3):375-387. doi:10.1093/jpepsy/jsz001
6. Murry VM, Kettrey HH, Berkel C, Inniss-Thompson MN. The Pathways for African American Success: Does Delivery Platform Matter in the Prevention of HIV Risk Vulnerability Among Youth? *J Adolesc Health*. Aug 2019b;65(2):255-261. doi:10.1016/j.jadohealth.2019.02.013
7. Murry VM, Berkel C, Liu N. The Closing Digital Divide: Delivery Modality and Family Attendance in the Pathways for African American Success (PAAS) Program. *Prev Sci*. Jul 2018;19(5):642-651. doi:10.1007/s11121-018-0863-z
