# Supplementary material for: Comparing Digital Versus Face-to-Face Delivery of Systemic Psychotherapy Interventions: Systematic Review and Meta-Analysis of Randomized Controlled Trials
Source: Interact J Med Res. 2025 Feb 24;14:e46441. doi: 10.2196/46441 (PMC11894358; doi:10.2196/46441)
Supplement: Multimedia Appendix 6 [file ijmr_v14i1e46441_app6.docx]

**Multimedia Appendix 6:** Reported statistics on face-to-face vs. digital delivery

**Table 1.** Reported statistics on face-to-face vs. digital delivery (N=754^a^)

| Trial | Publication | N (n F2F, n DD) | Outcome measures | Time points assessed |
| --- | --- | --- | --- | --- |
|  |  |  |  |  |
| BFST-D | Duke et al (2016)^1^ | 90 (44, 46) | Y-HHI, Y-CBQ, Y-AIS, P-HHI, P-CBQ, P-AIS, Y-DSMP, P-DSMP, HbA1c | Pre, post (4 weeks), follow-up (12 weeks) |
|  | Harris et al (2015)^2^ | 90 (44, 46) | Y-DSMP, P-DSMP, HbA1c | Pre, post (4 weeks), follow-up (12 weeks) |
|  | Freeman et al (2013)^3^ | 92 (only participants reporting WAI at post-test included=39, 32) | Y-WAI, P-WAI, HbA1c, Y-CBQ, P-CBQ, Y-DRFC, P-DRFC | After session 5, post-treatment (4 weeks); only post-treatment used for analysis |
|  | Riley et al (2015)^4^ | 90 (82 included across F2F and DD conditions) | CDI, HbA1c, Y-CBQ, Y-DFCS, Y-HHI, P-CBQ, P-DFCS, P-HHI | Pre, post (4 weeks), follow-up (12 weeks) |
| PAAS | Murry et al (2019a)^5^ | 421 (141, 141; 136 CC) | Primary outcomes: CSS, Y-OSFC, Y-FoC, Y-DQ, Y-CiC, P-OSFC, P-FoC, P-DQ, P-CiC, SURCS expanded, FoSC, CoRH, SIQS, AwDPS. Secondary outcomes: SRS, MFS | Pre, post (Mean months 14.5, SD 4.4), follow-up (Mean months 22.6, SD 3.7). Pre to post for primary parent and youth outcomes, pre to follow-up for secondary outcomes |
|  | Murry et al (2019b)^6^ | 412 (137, 138; 137 CC) | Y-AN/EaRE, Y-OSFC, Y-FoC, Y-DQ, Y-CiC, Y-REI, P-AN/EaRE, P-OSFC, P-FoC, P-DQ, P-CiC | Pre, post (6 months) |
|  | Murry et al (2018)^7^ | 412 (137, 138; 137 CC) | At least 1 session attended, number of sessions attended, retention at least 50% of sessions, retention across all sessions, drop out after 1 session | n/a |
| F-PST | Kurowski et al (2020)^8^ | 150 (34, 56; 60 SG) | Y-BRIEF GEC, P-BRIEF GEC Parent, SDQ | Pre, post (6 months), follow-up (9 months) |
|  | Wade et al (2019a)^9^ | 150 (34, 56; 60 SG) | CES-D, BSI | Pre, post (6 months), follow-up (9 months) |
|  | Wade et al (2019b)^10^ | 150 (34, 56; 60 SG) | Y-PedsQL, P-PedsQL, P-HBI, Y-HBI | Pre, post (6 months), follow-up (9 months) |
|  | Wade et al (2019c)^11^ | 150 (34, 56; 60 SG) | Y-PE, P-PE, Y-PB, P-PB, Y-OS, P-OS, adherence, BRIEF, SDQ | Pre, post (6 months), follow-up (9 months) |
| SUCCEAT | Truttmann et al (2020)^12^ | 102 (50, 52) | GHQ, EDSIS, SCL 90-R, BDI-II, STAI State, STAI Trait, CASK | Pre, post (3 months), follow-up (12 months) |

**Table 1.** (continued)

| Trial | Study | Statistical analyses | Results F2F vs. DD comparisons |
| --- | --- | --- | --- |
| BFST-D | Duke et al (2016)^1^ | ITT analysis with multiple imputation procedures to impute missing data; between groups repeated measures ANOVA | Between condition comparisons across timepoints: Y-HHI: *F*_1_=0.08, *P*=.78; Y-CBQ: *F*_1_=0.32, *P*=.57; Y-AIS: *F*_1_=3.87, *P*=.052; P-HHI: *F*_1_=0.98, *P*=.33; P-CBQ: *F*_1_=0.27, *P*=.60; P-AIS: *F*_1_=1.00, *P*=.32 |
|  | Harris et al (2015)^2^ | ITT analysis with pooled means to impute missing data; between groups repeated measures ANOVA | Between condition comparisons: Y-DSMP, P-DSMP, HbA1c: *F*_1_=0.09; *P*=0.77); absence of stat. sig. differences between conditions on outcome measures reported |
|  | Freeman et al (2013)^3^ | ITT analysis with mean score of items in specific factors used to impute missing data; independent samples t-tests | Between condition comparisons: Y-WAI (all subscales): *t*_69_=0.834^b^; P-WAI (all subscales): *t*_69_ = 0.528^b^ |
|  | Riley et al (2015)^4^ | ITT analysis with last observation forward imputation for missing data; no information on statistical test for F2F vs. DD comparison | Reported no statistically significant differences between conditions on measures of depressive symptoms and family processes |
| PAAS | Murry et al (2019a)^7^ | ITT analysis; full Information Maximum Likelihood imputation of missing data; structural equation modelling. Outcomes for Sexual Risk Survey and Monitoring the Future Scale combined due to low completion rates at follow-up. Two latent constructs used to assess two dimensions of parenting: General supportive parenting (i.e., caregiver support, open communication, frequency of communication) and parenting with respect to sensitive topics (i.e., racial socialization, communication about sex, and setting up clear rules about substance use). | Primary outcomes^c^: Latent construct general supportive parenting: Significant improvement for face-to-face delivery condition: β=.12, 95% CI .02-.20, *P*=.02. Latent construct parenting with respect to sensitive topics: Significant improvement for digital delivery condition: β=.30, 95% CI .09-.54, *P*=.03. SIQS: Significant decrease in score for digital delivery condition: β=.12, 95% CI .20-.01; *P*=.04. AwDPS: Significant increase in score in face-to-face condition: β=.16, 95% CI .06-.27; *P*=.002. Secondary outcomes^f^: SRS and MFS combined: Significant decrease in score in digital delivery condition: β=.17, 95% CI .31-.04; *P*= .04. |
|  | Murry et al (2019b)^6^ | ITT analysis; Standardized mean differences, reported as unadjusted effect sizes with confidence intervals | Y-REI: Significantly lower in the digital delivery modality condition compared to the face-to-face delivery modality condition (*d*=-0.36, 95% CI -.63--.10, *P*<.05). No other stat. sig. differences at p < .05 level. |
|  | Murry et al (2018)^7^ | ITT analysis, listwise deletion for missing data, simple linear regression | At least 1 session attended: Higher in digital delivery condition (71% vs. 57%, *F*_1277_=27. Number of sessions attended: Higher in digital delivery condition (Mean=4, SD 3 vs. Mean = 2, SD = 2). Retention at least 50% of sessions: Higher in digital delivery condition (67% vs. 45%, *χ*^2^_1_=14.05) and retained for all sessions (52% vs. 16%, *χ*^2^_1_=42.14). They were less likely to drop out after one session (1% vs. 7%, *χ*^2^_1_=6.18). |
| F-PST | Kurowski et al (2020)^8^ | ITT analysis; repeated-measures mixed models; effect sizes derived from mixed-model results and similar to Cohen *d*, adjusted *t* statistics for pairwise differences | All outcomes: Reported no statistically significant differences between conditions |
|  | Wade et al (2019a)^9^ | ITT analysis, generalized linear mixed models with repeated measures, Cohen *d* and *P* values for least square means by treatment group over time, post-hoc slope analyses | CES-D: *F*_2,131_=2.49, *P*=.087; BSI: Reported no statistically significant differences between conditions |
|  | Wade et al (2019b)^10^ | ITT analysis, generalized linear mixed models with repeated measures, Cohen *d* and *P* values for least square means by treatment group over time, post-hoc slope analyses | PedsQL at post: SG significantly higher than F2F (*d*=0.9, *P=*.019), DD significantly higher than F2F (*d*=0.54, *P=*.035); PedsQL at follow-up: SG significantly higher than F2F (*d*=0.58, *P=*.023); HBI cognitive at post: F2F significantly higher than SG (*d*=0.51, *P=*.048); HBI cognitive at follow-up: F2F significantly higher than SG (*d*=0.77, *P=*.003); HBI somatic at post: F2F significantly higher than SG (*d*=0.51, *P=*.048); HBI somatic at follow-up: F2F significantly higher than SG (*d*=0.54, *P=*.036). No other statistically significant differences between groups reported. |
|  | Wade et al (2019c)^11^ | ITT analysis, chi-square or Fisher exact test for relationship between predictors, sample characteristics, and outcome measures; t-tests for normally distributed data (Bonferroni-corrected where appropriate), Wilcoxon rank sum test for skewed data; Pearson correlation, Spearman correlation, Point biserial correlation for correlations between predictors, covariates, and outcome measures; McNemar statistic for differences in proportions; logistic regression, general linear modeling to examine factors associated with treatment preference and satisfaction; linear mixed models for interactions between treatment group and and treatment preference over time | P-PE: Higher scores for F2F condition compared to DD (Cohen *d=*0.67, 95% CI 0.10-1.15, *t=*-2.49, *P* < .043) and SG (Cohen *d*=1.18, 95% CI 0.56-1.62, *t*=-4.36, *P* < .001).P-OS: Higher scores for F2F condition compared to SG (Cohen *d*=0.63, 95% CI 0.09-1.11, *t*=-2.51, *P*=.040) |
| SUCCEAT | Truttmann et al (2020)^12^ | ITT analysis, expectation-maximization method to impute missing data; general linear mixed models, between groups repeated ANOVA | Time x Condition Interaction effects: GHQ: *F*=0.753, *P*=0.473; EDSIS: *F*=0.166, *P*=0.847; SCL 90-R: *F*=2.261, *P*=0.107; BDI-II: *F*=2.394, *P*=0.094; STAI State: *F*=3.312, *P*=0.039; STAI Trait: *F*=0.169, *P*=0.845; CASK: *F*=0.636, *P*=0.530 |

^a^: Sum of n at trial level. In case of inconsistencies in reported n across individual studies for each trial, the mode n was selected.

^b^: Degrees of freedom not provided in original study. Calculated by hand based on numbers of participants for both conditions

^c^: Relation between delivery condition and outcome variables reported only for outcomes singularly affected by one condition.

Abbreviations: F2F, face-to-face; DD, digital delivery; Y-HHI, Helping for Health Inventory, youth report; Y-CBQ, Conflict Behavior Questionnaire Short Form, youth report; Y-DFCS, Diabetes Family Conflict Scale, youth-report; Y-AIS, Acceptance of Illness Scale, youth report; P-HHI, Helping for Health Inventory, parent report; P-CBQ, Conflict Behavior Questionnaire Short Form, parent report; P-DFCS, Diabetes Family Conflict Scale, parent-report; CC, control condition;P-AIS, Acceptance of Illness Scale, parent report; Y-DSMP, Diabetes Self-Management Profile, youth report; P-DSMP, Diabetes Self-Management Profile, parent report; HbA1c, hemoglobin A1c; Y-WAI, Working Alliance Inventory (Client), youth report; P-WAI, Working Alliance Inventory (Client), parent report; Y-DRFC, Diabetes Responsibility and Family Conflict Scale, youth report; P-DRFC, Diabetes Responsibility and Family Conflict Scale, parent report; CDI, Child Depression Inventory; CSS, Caregiver Support scale subscale, Multidimensional coping Inventory; Y-OSFC, Discussion Quality Scale, youth report; Y-FoC, Frequency of Conversation, youth report; Y-DQ, Parent-Youth Discussion Quality, youth report; Y-CiC, Conflicted an Ineffective Communication, youth report; P-OSFC, Discussion Quality Scale, parent report; P-FoC, Frequency of Conversation, parent report; P-DQ, Parent-Youth Discussion Quality, parent report; P-CiC, Conflicted an Ineffective Communication, parent report; SG, self-guided delivery; SURCS, Substance Use Rules Communication Scale from Strengthening Families, expanded version; FoSC, Frequency of Sexual Communication Scale; CoRH, Celebration of racial heritage subscale, Racial Socialization Scale; SIQS, Substance Intention Questions Scale; AwDPS, Affiliation with Deviant Peers Scale; SRS, Sexual Risk Survey; MFS, Monitoring the Future Scale; Y-AN/EaRE, Articulated Norms and Expectations about Risk Engagement, youth report; P-AN/EaRE, Articulated Norms and Expectations about Risk Engagement, parent report; Y-REI, Youth Risk Engagement Intentions; Y-BRIEF GEC, Behaviour Rating Inventory of Executive Function. Global Executive Functioning; P-BRIEF GEC, Behaviour Rating Inventory of Executive Function. Global Executive Functioning; SDQ, Strengths and Difficulties Questionnaire; CES-D, Center for Epidemiological Studies Depression Scale; BSI, Brief Symptom Inventory; PedsQL, Paediatric Quality of Life; HBI, Health Behaviour Inventory; P-PE, Programme Evaluation, parent reported; Y-PE, Programme Evaluation, youth reported; P-PB, Perceived Benefit, parent reported; P-OS, Overall Satisfaction, parent reported;Y-PB, Perceived Benefit, youth reported; Y-OS, Overall Satisfaction, youth reported; P, Parent reported; Y, Youth reported; GHQ, General Health Questionnaire; EDSIS, Eating Disorder Symptom Impact Scale; SCL 90-R, Symptom Checklist Global Severity Index; BDI-II, Beck Depression Inventory; STAI State, State and Trait Anxiety Inventory, State subscale; STAI Trait, Trait subscale; CASK, Caregiver Skills scale;

# References

1. Duke DC, Wagner DV, Ulrich J, Freeman KA, Harris MA. Videoconferencing for Teens With Diabetes: Family Matters. *J Diabetes Sci Technol*. Jul 2016;10(4):816-23. doi:10.1177/1932296816642577

2. Harris MA, Freeman KA, Duke DC. Seeing Is Believing: Using Skype to Improve Diabetes Outcomes in Youth. *Diabetes Care*. Aug 2015;38(8):1427-34. doi:10.2337/dc14-2469

3. Freeman KA, Duke DC, Harris MA. Behavioral health care for adolescents with poorly controlled diabetes via Skype: does working alliance remain intact? *J Diabetes Sci Technol*. May 1 2013;7(3):727-35. doi:10.1177/193229681300700318

4. Riley AR, Duke DC, Freeman KA, Hood KK, Harris MA. Depressive Symptoms in a Trial Behavioral Family Systems Therapy for Diabetes: A Post Hoc Analysis of Change. *Diabetes Care*. Aug 2015;38(8):1435-40. doi:10.2337/dc14-2519

5. Murry VM, Berkel C, Inniss-Thompson MN, Debreaux ML. Pathways for African American Success: Results of Three-Arm Randomized Trial to Test the Effects of Technology-Based Delivery for Rural African American Families. *J Pediatr Psychol*. Apr 1 2019a;44(3):375-387. doi:10.1093/jpepsy/jsz001

6. Murry VM, Kettrey HH, Berkel C, Inniss-Thompson MN. The Pathways for African American Success: Does Delivery Platform Matter in the Prevention of HIV Risk Vulnerability Among Youth? *J Adolesc Health*. Aug 2019b;65(2):255-261. doi:10.1016/j.jadohealth.2019.02.013

7. Murry VM, Berkel C, Liu N. The Closing Digital Divide: Delivery Modality and Family Attendance in the Pathways for African American Success (PAAS) Program. *Prev Sci*. Jul 2018;19(5):642-651. doi:10.1007/s11121-018-0863-z

8. Kurowski BG, Taylor HG, McNally KA, et al. Online Family Problem-Solving Therapy (F-PST) for Executive and Behavioral Dysfunction After Traumatic Brain Injury in Adolescents: A Randomized, Multicenter, Comparative Effectiveness Clinical Trial. *J Head Trauma Rehabil*. May/Jun 2020;35(3):165-174. doi:10.1097/htr.0000000000000545

9. Wade SL, Cassedy AE, McNally KA, et al. A Randomized Comparative Effectiveness Trial of Family-Problem-Solving Treatment for Adolescent Brain Injury: Parent Outcomes From the Coping with Head Injury through Problem Solving (CHIPS) Study. *J Head Trauma Rehabil*. Nov/Dec 2019a;34(6):E1-e9. doi:10.1097/htr.0000000000000487

10. Wade SL, Cassedy AE, Sklut M, et al. The Relationship of Adolescent and Parent Preferences for Treatment Modality With Satisfaction, Attrition, Adherence, and Efficacy: The Coping With Head Injury Through Problem-Solving (CHIPS) Study. *J Pediatr Psychol*. Apr 1 2019b;44(3):388-401. doi:10.1093/jpepsy/jsy087

11. Wade SL, Cassedy AE, Taylor HG, et al. Adolescent quality of life following family problem-solving treatment for brain injury. *J Consult Clin Psychol*. Nov 2019c;87(11):1043-1055. doi:10.1037/ccp0000440

12. Truttmann S, Philipp J, Zeiler M, et al. Long-Term Efficacy of the Workshop Vs. Online SUCCEAT (Supporting Carers of Children and Adolescents with Eating Disorders) Intervention for Parents: A Quasi-Randomised Feasibility Trial. *J Clin Med*. Jun 18 2020;9(6)doi:10.3390/jcm9061912
