# Supplementary material for: Comparing Digital Versus Face-to-Face Delivery of Systemic Psychotherapy Interventions: Systematic Review and Meta-Analysis of Randomized Controlled Trials
Source: Interact J Med Res. 2025 Feb 24;14:e46441. doi: 10.2196/46441 (PMC11894358; doi:10.2196/46441)
Supplement: Multimedia Appendix 7 [file ijmr_v14i1e46441_app7.docx]

**Multimedia Appendix 7:** Characteristics of included articles

**Table 1.** Characteristics of included studies (N=754^a^)

| Trial | Publication | Country | RCT Design | N (n F2F, n DD) | Mean youth age in years (SD) | Youth Male (%) | Youth Feature | Mean parent age in years (SD) | Parent Female (%) |
| --- | --- | --- | --- | --- | --- | --- | --- | --- | --- |
|  |  |  |  |  |  |  |  |  |  |
| BFST-D | Duke et al (2016)^1^ | USA | Parallel, 2 arm | 90 (46, 44) | 15.02 (1.75) | 55 (61.1) | HbA1c > 9% | – | 76.7 |
|  | Harris et al (2015)^2^ | USA | Parallel, 2 arm | 90 (46, 44) | 14.9^e^ (1.7) | 55 (61.1)^e^ | HbA1c > 9% | – | – |
|  | Freeman et al (2013)^3^ | USA | Parallel, 2 arm | 92 (45, 47) | 15.1^c^ | 42 (45.7)^e^ | HbA1c > 9% | – | – |
|  | Riley et al (2015)^4^ | USA | Parallel, 2 arm | 82^d^ | 14.1 | 55 (61.1) | HbA1c > 9% | – | – |
| PAAS | Murry et al (2019a)^5^ | USA | Parallel, 3 arm | 421 (141, 141, 136 CC) | - | 195^e^ (46.0) | PRB | 40 | 84 |
|  | Murry et al (2019b)^6^ | USA | Parallel, 3 arm | 412 (137, 138, 137 CC) | 11.0 | 191^e^ (46.0) | PRB | 40 | 84.3 |
|  | Murry et al (2018)^7^ | USA | Parallel, 3 arm | 412 (137, 138, 137 CC) | 11.4 | 191 (46.4) | PRB | 40 | – |
| F-PST | Kurowski et al (2020)^8^ | USA | Quasi, 3 arm | 150 (34, 56, 60 SG) | 16.5 (1.1) | 96 (64.0) | TBI | – | – |
|  | Wade et al (2019a)^9^ | USA | Quasi, 3 arm | 150 (34, 56, 60 SG) | 16.5 (1.1) | 96 (64.0) | TBI | – | 84.7 |
|  | Wade et al (2019c)^10^ | USA | Quasi, 3 arm | 150 (34, 56, 60 SG) | 16.5 (1.1) | 96 (64.0) | TBI | – | – |
|  | Wade et al (2019b)^11^ | USA | Quasi, 3 arm | 150 (34, 56, 60 SG) | 15.5 (1.5)^f^ | 96 (64.0) | TBI | 45.8 (8.5) | 84.7 |
| SUCCEAT | Truttmann et al (2020)^12^ | AT | Quasi, time sequence, 2 arm | 102 (50, 52) | 14.9 (1.9) | 9 (8.9) | AN | 47.18 (6.90)^e^ | 86 |

**Table 1. (**continued)

| Trial | DI | F2FI | Provider | Fidelity | Average number of sessions (SD) | Recipient(s) |
| --- | --- | --- | --- | --- | --- | --- |
|  |  |  |  |  |  |  |
| BFST-D | VC | Clinic | Psychology MA & PhD | Training, supervision, cross delivery | 5.8 (3.3) | Youth & parent^b^ |
|  | VC | Clinic | Psychology MA & PhD | Training, supervision, cross delivery | 5.8 (3.3) | Youth & parent |
|  | VC | Clinic | Psychology intern & PhD | Training, supervision, cross delivery | – | Youth & parent |
|  | VC | Clinic | Psychology MA & PhD | Training, supervision, cross delivery | 6.3 (3.4) | Youth & parent |
| PAAS | II | GS | Community facilitators | Training, curriculum, recorded & fidelity scored (>80%) | – | Youth & parent, sibling(s) |
|  | II | GS | Community facilitators | Training, manual | – | Youth & parent, sibling(s) |
|  | II | GS | Community facilitators | Training, curriculum, recorded & fidelity scored (>80%) | 4.0 (3.0) | Youth & parent, sibling(s) |
| F-PST | VC+OP^g^ | Clinic | Clinical Psychologists | Training, manual, supervision, checklist | 6.3 (2.6) | Youth and family |
|  | VC+OP^g^ | Clinic | Clinical Psychologists | Training, manual, supervision | 6.3 (2.6) | Youth and family |
|  | VC+OP^g^ | Clinic | Clinical Psychologists | Training, manual, supervision, checklist | 6.3 (2.6) | Youth and family |
|  | VC+OP^g^ | Clinic | Clinical Psychologists | Training, manual, supervision | 6.3 (2.6) | Youth and family |
| SUCCEAT | OP | GWS | Psychiatrist & Medical Doctor | Specific training on parts of intervention content, manual, work in the field | 6.5 (2.0) | Parent |

^a^ Sum of n at the trial level. In case of inconsistencies in reported n across individual publications for each trial, the mode n was selected.

^b^ parent: primary caregiver

^c^ calculated based on mean ages, sample sizes and SDs provided

^d^ only participants who completed CDI measure (only age > 18 eligible)

^e^ calculated based on percentages and overall sample size provided

^f^ most likely a clerical mistake in reporting

^g^ two distinct digital delivery conditions: i) video conferencing plus online program, and ii) online program

Abbreviations: F2F, face-to-face delivery; DD, therapist-guided digital delivery; DI, digital implementation; F2FI, face-to-face implementation; BFST-D, Behavioral Family Systems Therapy for Diabetes; USA, United States of America; HbA1C, hemoglobin A1c; VC, videoconferencing; PAAS, Pathways for African American Success; F-PST, Family Problem Solving Therapy; CC, control condition; PRB, potential risk-behavior; II, interactive interface; GS, group session; SG, self-guided digital delivery; TBI, traumatic brain injury; SUCCEAT, Supporting Carers of Children and Adolescents with Eating Disorders in Austria; AT, Austria; AN, anorexia nervosa; OP, online program; GWS, group workshop

**References**

1. Duke DC, Wagner DV, Ulrich J, Freeman KA, Harris MA. Videoconferencing for Teens With Diabetes: Family Matters. *J Diabetes Sci Technol*. Jul 2016;10(4):816-23. doi:10.1177/1932296816642577
2. Harris MA, Freeman KA, Duke DC. Seeing Is Believing: Using Skype to Improve Diabetes Outcomes in Youth. *Diabetes Care*. Aug 2015;38(8):1427-34. doi:10.2337/dc14-2469
3. Freeman KA, Duke DC, Harris MA. Behavioral health care for adolescents with poorly controlled diabetes via Skype: does working alliance remain intact? *J Diabetes Sci Technol*. May 1 2013;7(3):727-35. doi:10.1177/193229681300700318
4. Riley AR, Duke DC, Freeman KA, Hood KK, Harris MA. Depressive Symptoms in a Trial Behavioral Family Systems Therapy for Diabetes: A Post Hoc Analysis of Change. *Diabetes Care*. Aug 2015;38(8):1435-40. doi:10.2337/dc14-2519
5. Murry VM, Berkel C, Inniss-Thompson MN, Debreaux ML. Pathways for African American Success: Results of Three-Arm Randomized Trial to Test the Effects of Technology-Based Delivery for Rural African American Families. *J Pediatr Psychol*. Apr 1 2019a;44(3):375-387. doi:10.1093/jpepsy/jsz001
6. Murry VM, Kettrey HH, Berkel C, Inniss-Thompson MN. The Pathways for African American Success: Does Delivery Platform Matter in the Prevention of HIV Risk Vulnerability Among Youth? *J Adolesc Health*. Aug 2019b;65(2):255-261. doi:10.1016/j.jadohealth.2019.02.013
7. Murry VM, Berkel C, Liu N. The Closing Digital Divide: Delivery Modality and Family Attendance in the Pathways for African American Success (PAAS) Program. *Prev Sci*. Jul 2018;19(5):642-651. doi:10.1007/s11121-018-0863-z
8. Kurowski BG, Taylor HG, McNally KA, et al. Online Family Problem-Solving Therapy (F-PST) for Executive and Behavioral Dysfunction After Traumatic Brain Injury in Adolescents: A Randomized, Multicenter, Comparative Effectiveness Clinical Trial. *J Head Trauma Rehabil*. May/Jun 2020;35(3):165-174. doi:10.1097/htr.0000000000000545
9. Wade SL, Cassedy AE, McNally KA, et al. A Randomized Comparative Effectiveness Trial of Family-Problem-Solving Treatment for Adolescent Brain Injury: Parent Outcomes From the Coping with Head Injury through Problem Solving (CHIPS) Study. *J Head Trauma Rehabil*. Nov/Dec 2019a;34(6):E1-e9. doi:10.1097/htr.0000000000000487
10. Wade SL, Cassedy AE, Taylor HG, et al. Adolescent quality of life following family problem-solving treatment for brain injury. *J Consult Clin Psychol*. Nov 2019c;87(11):1043-1055. doi:10.1037/ccp0000440
11. Wade SL, Cassedy AE, Sklut M, et al. The Relationship of Adolescent and Parent Preferences for Treatment Modality With Satisfaction, Attrition, Adherence, and Efficacy: The Coping With Head Injury Through Problem-Solving (CHIPS) Study. *J Pediatr Psychol*. Apr 1 2019b;44(3):388-401. doi:10.1093/jpepsy/jsy087
12. Truttmann S, Philipp J, Zeiler M, et al. Long-Term Efficacy of the Workshop Vs. Online SUCCEAT (Supporting Carers of Children and Adolescents with Eating Disorders) Intervention for Parents: A Quasi-Randomised Feasibility Trial. *J Clin Med*. Jun 18 2020;9(6)doi:10.3390/jcm9061912
