# Supplementary material for: Comparing Digital Versus Face-to-Face Delivery of Systemic Psychotherapy Interventions: Systematic Review and Meta-Analysis of Randomized Controlled Trials
Source: Interact J Med Res. 2025 Feb 24;14:e46441. doi: 10.2196/46441 (PMC11894358; doi:10.2196/46441)
Supplement: Multimedia Appendix 8 [file ijmr_v14i1e46441_app8.docx]

**Multimedia Appendix 8:** Outcome measures overview

| Outcome | Abbreviation | Full name | Used in publication | Function | Quality | No. of items | Delivery | Scoring | Reference |
| --- | --- | --- | --- | --- | --- | --- | --- | --- | --- |
|  |  |  |  |  |  |  |  |  |  |
| Primary | HbA1c | HbA1c assay | Harris et al., 2015^1^ | Determine percentage of glycated hemoglobin | Valid and reliable | n/a | Measured by clinic nursing staff using Siemens/Bayer DCA 2000 analyzer | Percentage | Tamborlane WV, Kollman C, Steffes MW, et al. Comparison of fingerstick hemoglobin A1c levels assayed by DCA 2000 with the DCCT/EDIC central laboratory assay: results of a Diabetes Research in Children Network (DirecNet) Study. *Pediatr Diabetes*. 2005;6(1):13-16. doi:10.1111/j.1399-543X.2005.00088.x |
|  | Y-DSMP | Diabetes Self-Management Profile, youth report | Harris et al., 2015 ^1^ | Assess adherence to diabetes management | Valid and reliable | 25 | Semi-structured interview | Higher scores indicate higher levels of adherence | Harris MA, Wysocki T, Sadler M, et al. Validation of a structured interview for the assessment of diabetes self-management. *Diabetes Care*. 2000;23(9):1301-1304. doi:10.2337/diacare.23.9.1301 |
|  | P-DSMP | Diabetes Self-Management Profile, parent report | Harris et al., 2015 ^1^ | Assess adherence to diabetes management | Valid and reliable | 25 | Semi-structured interview | Higher scores indicate higher levels of adherence | Harris MA, Wysocki T, Sadler M, et al. Validation of a structured interview for the assessment of diabetes self-management. *Diabetes Care*. 2000;23(9):1301-1304. doi:10.2337/diacare.23.9.1301 |
|  | Y-REI | Youth Risk Engagement Intentions | Murry et al., 2019b^2^ | Assess intentions to smoke cigarettes, drink alcohol, use marijuana, and use other drugs in next year | Cronbach a > .86 at pre and post-tests; probably adapted from custom scale | 8 | Questionnaire administered through computer-assisted interview | Higher scores indicate increased intentions to engage in risk behaviours | Gerrard M, Gibbons FX, Reis-Bergan M, Trudeau L, Vande Lune LS, Buunk B. Inhibitory effects of drinker and nondrinker prototypes on adolescent alcohol consumption. *Health Psychol*. 2002;21(6):601-609. doi:10.1037//0278-6133.21.6.601  Gerrard M, Gibbons FX, Zhao L, Russell DW, Reis-Bergan M. The effect of peers' alcohol consumption on parental influence: a cognitive mediational model. *J Stud Alcohol Suppl*. 1999;13:32-44. doi:10.15288/jsas.1999.s13.32 |
|  | BRIEF, parent report | Behaviour Rating Inventory of Executive Function. Global Executive Functioning, Behaviour Regulation Index, Metacognition Index, parent format | Kurowski et al., 2020^3^ | Assesses adolescent’s executive function in daily life | Valid and reliable | 86 | Paper and pencil, Online administration and scoring via PARiConnect | Higher scores indicate better Executive Functioning | Roth RM, Isquith PK, Gioia GA. BRIEF-A: Behavior Rating Inventory  of Executive Function—Adult Version. Lutz, FL: Psychological  Assessment Resources, Inc; 2005. |
|  | BRIEF, parent report | Behaviour Rating Inventory of Executive Function. Global Executive Functioning, Behaviour Regulation Index, Metacognition Index, adolescent format | Kurowski et al., 2020^3^ | Assesses adolescent’s executive function in daily life | Valid and reliable | 86 | Paper and pencil, Online administration and scoring via PARiConnect | Higher scores indicate better Executive Functioning | Gioia G, Isquith PK, Guy SC, Kenworthy L. BRIEF: Behavior  Rating Inventory of Executive Function. Lutz, FL: Psychological Assessment  Resources, Inc; 2000. |
|  | SDQ | Strengths and Difficulties Questionnaire. Externalizing and Internalizing combined | Kurowski et al., 2020^3^ | Parent rating of child externalizing and internalizing problem behaviour | Valid and reliable | 25 | Self-report | Higher scores are associated with more  difficulties, with Total scores of 17 or greater indicating  high difficulties. | Bourdon KH, Goodman R, Rae DS, Simpson G, Koretz DS.  The strengths and difficulties questionnaire: U.S. normative data  and psychometric properties. J Am Acad Child Adolesc Psychiatry.  2005;44(6):557–564 |
|  | GHQ | General Health Questionnaire | Truttman et al., 2020^4^ | Assesses the level of parental  psychological distress | Valid and reliable | 12 | Self-report | Level of psychological distress rated on 4-point scale, where higher scores indicate higher levels of distress. | Linden, M.; Maier, W.; Achberger, M.; Herr, R.; Helmchen, H.; Benkert, O. Psychische erkrankungen  und ihre behandlung in allgemeinarztpraxen in Deutschland: Ergebnisse aus einer studie der  Weltgesundheitsorganisation (WHO). [Psychological disorders and their treatment in general practice  in Germany: Results of a WHO study.]. Nervenarzt 1996, 67, 205–215. |
| Secondary | Y-HHI | Helping for Health Inventory, youth report | Duke et al.,, 2016^5^ | Assesses maladaptive interactions between caregiver and adolescents with regards to assistance in diabetes management (“miscarried helping”) | Valid and reliable | 15 | Self-report | Frequency of occurrence of events rated on 5-point scale, where higher scores indicate more frequent occurrence and higher frequency of miscarried helping | Harris MA, Antal H, Oelbaum R, Buckloh LM, White NH, Wysocki T. Good intentions gone awry: Assessing parental “miscarried helping” in diabetes. Families, Systems & Health. 2008;26(4):393–403 |
|  | P-HHI | Helping for Health Inventory, parent report | Duke et al.,, 2016^5^ | Assesses maladaptive interactions between caregiver and adolescents with regards to assistance in diabetes management (“miscarried helping”) | Valid and reliable | 15 | Self-report |  | Harris MA, Antal H, Oelbaum R, Buckloh LM, White NH, Wysocki T. Good intentions gone awry: Assessing parental “miscarried helping” in diabetes. Families, Systems & Health. 2008;26(4):393–403 |
|  | Y-CBQ | Conflict Behavior Questionnaire Short Form, youth report | Duke et al.,, 2016^5^ | Assesses parent-adolescent conflict in terms of caregiver behavior | Valid and reliable | 20 | Self-report | True/false statements; higher scores indicate more conflict | Prinz RJ, Foster S, Kent RN, O'Leary KD. Multivariate assessment of conflict in distressed and nondistressed mother-adolescent dyads. *J Appl Behav Anal*. 1979;12(4):691-700. doi:10.1901/jaba.1979.12-691 |
|  | P-CBQ | Conflict Behavior Questionnaire Short Form, parent report | Duke et al.,, 2016^5^ | Assesses parent-adolescent conflict in terms of adolescent behavior | Valid and reliable | 20 | Self-report | True/false statements; higher scores indicate more conflict | Prinz RJ, Foster S, Kent RN, O'Leary KD. Multivariate assessment of conflict in distressed and nondistressed mother-adolescent dyads. *J Appl Behav Anal*. 1979;12(4):691-700. doi:10.1901/jaba.1979.12-691 |
|  | Y-AIS | Acceptance of Illness Scale, youth report | Duke et al.,, 2016^5^ | Assesses extent to which respondent accepts health condition without negative feelings or responses | Valid and reliable | 8 | No information, but likely self-report | Agreement to statements on 5-point scale, where higher scores indicate higher agreement and increased acceptance of illness | Felton BJ, Revenson TA, Hinrichsen GA. Stress and coping in the explanation of psychological adjustment among chronically ill adults. *Soc Sci Med*. 1984;18(10):889-898. doi:10.1016/0277-9536(84)90158-8 |
|  | P-AIS | Acceptance of Illness Scale, parent report | Duke et al.,, 2016^5^ | Assesses extent to which adolescent accepts health condition without negative feelings or responses | Valid and reliable | 8 | No information, but likely self-report | Agreement to statements on 5-point scale, where higher scores indicate higher agreement and increased acceptance of illness | Felton BJ, Revenson TA, Hinrichsen GA. Stress and coping in the explanation of psychological adjustment among chronically ill adults. *Soc Sci Med*. 1984;18(10):889-898. doi:10.1016/0277-9536(84)90158-8 |
|  | CDI | Child Depression Inventory | Riley et al., 2015^6^ | Evaluates severity of depressive symptoms in children and adolescents | Valid and reliable | 27 | No information, but likely self-report | Agreement to statements on 3-point scale, where higher scores indicate higher agreement. Scores from 13 and higher indicate elevated sub-clinical depressive symptoms, 19 and higher indicate clinical depressive symptoms | Kovacs M., Beck A.T. An empirical-clinical approach toward a definition of childhood depression. *Depression in childhood: Diagnosis, treatment, and conceptual models.* 1977:1–25. |
|  | Y-DFCS | Diabetes Family Conflict Scale, youth-report | Riley et al., 2015^6^ | Assesses specific diabetes-related family conflict | Valid and reliable | 19 | Self-report | 3-point scale where higher scores indicate higher levels of diabetes-related family conflict | Hood KK, Butler DA, Anderson BJ, Laffel LM. Updated and revised diabetes family confliict scale. Diabetes Care 2007;30:1764–1769 |
|  | P-DFCS | Diabetes Family Conflict Scale, parent-report | Riley et al., 2015^6^ | Assesses specific diabetes-related family conflict | Valid and reliable | 19 | Self-report | 3-point scale where higher scores indicate higher levels of diabetes-related family conflict | Hood KK, Butler DA, Anderson BJ, Laffel LM. Updated and revised diabetes family conflict scale. Diabetes Care 2007;30:1764–1769 |
|  | CSS | Caregiver Support scale subscale, Multidimensional coping Inventory | Murry et al., 2019a^7^ | Assesses supportiveness of relationship with adolescents | Valid and reliable | 4 | Questionnaire administered through computer-assisted interview | Agreement to statements on 5-point scale, where higher scores indicate higher agreement and higher levels of support | Carver CS, Scheier MF, Weintraub JK. Assessing coping strategies: a theoretically based approach. *J Pers Soc Psychol*. 1989;56(2):267-283. doi:10.1037//0022-3514.56.2.267 |
|  | Y-OSFC | Discussion Quality Scale, youth report | Murry et al., 2019a^7^Murry et al., 2019b^2^ | Open, supportive family communication, discretely reported for 3 dimensions (FoC, DQ, CiC) | Unclear, not unambiguously identifiable from provided references | 12 | Questionnaire administered through computer-assisted interview | See subscale levels (FoC, DQ, CiC) | McBride Murry V, Brody GH, McNair LD, et al. Parental Involvement Promotes Rural  African American Youths’ Self-Pride and Sexual Self-Concepts. *Journal of Marriage and Family.* 2005;67(3):627-642. doi:<https://doi.org/10.1111/j.1741-3737.2005.00158.x>  Ge X, Brody GH, Conger RD, Simons RL, Murry VM. Contextual amplification of pubertal transition effects on deviant peer affiliation and externalizing behavior among African American children. *Dev Psychol*. 2002;38(1):42-54. doi:10.1037//0012-1649.38.1.42 |
|  | P-OSFC | Discussion Quality Scale, parent report | Murry et al., 2019a^7^Murry et al., 2019b^2^ | Open, supportive family communication, discretely reported for 3 dimensions (FoC, DQ, CiC) | Unclear, not unambiguously identifiable from provided references | 12 | Questionnaire administered through computer-assisted interview | See subscale levels (FoC, DQ, CiC) | McBride Murry V, Brody GH, McNair LD, et al. Parental Involvement Promotes Rural  African American Youths’ Self-Pride and Sexual Self-Concepts. *Journal of Marriage and Family.* 2005;67(3):627-642. doi:<https://doi.org/10.1111/j.1741-3737.2005.00158.x>  Ge X, Brody GH, Conger RD, Simons RL, Murry VM. Contextual amplification of pubertal transition effects on deviant peer affiliation and externalizing behavior among African American children. *Dev Psychol*. 2002;38(1):42-54. doi:10.1037//0012-1649.38.1.42 |
|  | Y-FoC | Frequency of Conversation, youth report | Murry et al., 2019a^7^Murry et al., 2019b^2^ | Assesses frequency of conversations between caregiver and adolescent about school, friend choices, drugs, alcohol, and sex | Cronbach a > .62 at pre- and post-tests; unclear reliability and validity | 4 | Questionnaire administered through computer-assisted interview | Possible scores from 0 to 12. Higher scores indicate more frequent conversations | McBride Murry V, Brody GH, McNair LD, et al. Parental Involvement Promotes Rural  African American Youths’ Self-Pride and Sexual Self-Concepts. *Journal of Marriage and Family.* 2005;67(3):627-642. doi:<https://doi.org/10.1111/j.1741-3737.2005.00158.x>  Ge X, Brody GH, Conger RD, Simons RL, Murry VM. Contextual amplification of pubertal transition effects on deviant peer affiliation and externalizing behavior among African American children. *Dev Psychol*. 2002;38(1):42-54. doi:10.1037//0012-1649.38.1.42 |
|  | P-FoC | Frequency of Conversation, parent report | Murry et al., 2019a^7^Murry et al., 2019b^2^ | Assesses frequency of conversations between caregiver and adolescent about school, friend choices, drugs, alcohol, and sex | Cronbach a > .62 at pre- and post-tests; unclear reliability and validity | 4 | Questionnaire administered through computer-assisted interview | Possible scores from 0 to 12. Higher scores indicate more frequent conversations | McBride Murry V, Brody GH, McNair LD, et al. Parental Involvement Promotes Rural  African American Youths’ Self-Pride and Sexual Self-Concepts. *Journal of Marriage and Family.* 2005;67(3):627-642. doi:<https://doi.org/10.1111/j.1741-3737.2005.00158.x>  Ge X, Brody GH, Conger RD, Simons RL, Murry VM. Contextual amplification of pubertal transition effects on deviant peer affiliation and externalizing behavior among African American children. *Dev Psychol*. 2002;38(1):42-54. doi:10.1037//0012-1649.38.1.42 |
|  | Y-DQ | Parent-Youth Discussion Quality, youth report | Murry et al., 2019a^7^Murry et al., 2019b^2^ | Assesses extent to which tone, frequency, and quality of conversations between caregivers and adolescents about school, friends, alcohol/drugs, and sex were “open, supportive, allowing each to share [their] side of the issue” | Cronbach a > .65 at pre- and post-tests; unclear reliability and validity | 4 | Questionnaire administered through computer-assisted interview | Possible scores from 0 to 16. Higher scores indicate higher discussion quality | McBride Murry V, Brody GH, McNair LD, et al. Parental Involvement Promotes Rural  African American Youths’ Self-Pride and Sexual Self-Concepts. *Journal of Marriage and Family.* 2005;67(3):627-642. doi:<https://doi.org/10.1111/j.1741-3737.2005.00158.x>  Ge X, Brody GH, Conger RD, Simons RL, Murry VM. Contextual amplification of pubertal transition effects on deviant peer affiliation and externalizing behavior among African American children. *Dev Psychol*. 2002;38(1):42-54. doi:10.1037//0012-1649.38.1.42 |
|  | P-DQ | Parent-Youth Discussion Quality, parent report | Murry et al., 2019a^7^Murry et al., 2019b^2^ | Assesses extent to which tone, frequency, and quality of conversations between caregivers and adolescents about school, friends, alcohol/drugs, and sex were “open, supportive, allowing each to share [their] side of the issue” | Cronbach a > .73 at pre- and post-tests; unclear reliability and validity | 4 | Questionnaire administered through computer-assisted interview | Possible scores from 0 to 16. Higher scores indicate higher discussion quality | McBride Murry V, Brody GH, McNair LD, et al. Parental Involvement Promotes Rural  African American Youths’ Self-Pride and Sexual Self-Concepts. *Journal of Marriage and Family.* 2005;67(3):627-642. doi:<https://doi.org/10.1111/j.1741-3737.2005.00158.x>  Ge X, Brody GH, Conger RD, Simons RL, Murry VM. Contextual amplification of pubertal transition effects on deviant peer affiliation and externalizing behavior among African American children. *Dev Psychol*. 2002;38(1):42-54. doi:10.1037//0012-1649.38.1.42 |
|  | Y-CiC | Conflicted an Ineffective Communication, youth report | Murry et al., 2019a^7^Murry et al., 2019b^2^ | Assesses relative contributions of caregivers and adolescents to discussions about school, friends, alcohol/drugs, and sex as well as how often discussions become arguments. | Cronbach a > .74.  Modified version of Ineffective Arguing Inventory Scale. Scale not unambiguously identifiable from provided reference. | Probably 4 | Questionnaire administered through computer-assisted interview | Possible scores from 0 to 16. Higher scores indicate lower occurrences of arguments | McBride Murry V, Brody GH, McNair LD, et al. Parental Involvement Promotes Rural  African American Youths’ Self-Pride and Sexual Self-Concepts. *Journal of Marriage and Family.* 2005;67(3):627-642. doi:<https://doi.org/10.1111/j.1741-3737.2005.00158.x>  Ge X, Brody GH, Conger RD, Simons RL, Murry VM. Contextual amplification of pubertal transition effects on deviant peer affiliation and externalizing behavior among African American children. *Dev Psychol*. 2002;38(1):42-54. doi:10.1037//0012-1649.38.1.42  Brody GH, Murry VM, Kim S, Brown AC. Longitudinal pathways to competence and psychological adjustment among African American children living in rural single-parent households. *Child Dev*. 2002;73(5):1505-1516. doi:10.1111/1467-8624.00486 |
|  | P-CiC | Conflicted an Ineffective Communication, parent report | Murry et al., 2019a^7^Murry et al., 2019b^2^ | Assesses relative contributions of caregivers and adolescents to discussions about school, friends, alcohol/drugs, and sex as well as how often discussions become arguments. | Cronbach a > .74.  Modified version of Ineffective Arguing Inventory Scale. Scale not unambiguously identifiable from provided reference. | Probably 4 | Questionnaire administered through computer-assisted interview | Possible scores from 0 to 16. Higher scores indicate lower occurrences of arguments | McBride Murry V, Brody GH, McNair LD, et al. Parental Involvement Promotes Rural  African American Youths’ Self-Pride and Sexual Self-Concepts. *Journal of Marriage and Family.* 2005;67(3):627-642. doi:<https://doi.org/10.1111/j.1741-3737.2005.00158.x>  Ge X, Brody GH, Conger RD, Simons RL, Murry VM. Contextual amplification of pubertal transition effects on deviant peer affiliation and externalizing behavior among African American children. *Dev Psychol*. 2002;38(1):42-54. doi:10.1037//0012-1649.38.1.42 |
|  | SURCS expanded | Substance Use Rules Communication Scale from Strengthening Families, expanded version | Murry et al., 2019a^7^ | Assesses caregivers’ explanations of substance use rules and consequences of violations of rules | Cronbach a > 90 at pre- and post-tests. Unclear reliability and validity of original version. | 9 | Questionnaire administered through computer-assisted interview | Agreement to statements on 5-point scale, where higher scores indicate higher levels of positive communication about substance use rules and consequences | Spoth R, Redmond C, Shin C. Direct and indirect latent-variable parenting outcomes of two universal family-focused preventive interventions: extending a public health-oriented research base. *J Consult Clin Psychol*. 1998;66(2):385-399. doi:10.1037//0022-006x.66.2.385 |
|  | FoSC | Frequency of Sexual Communication Scale | Murry et al., 2019a^7^ | Assess frequency of conversations about different topics concerning sexual behaviour | Cronbach a > .95 at pre- and post-tests. Article referenced reports Cronbach a > .73, but provides insufficient evidence of validity and reliability. | 8 | Questionnaire administered through computer-assisted interview | Frequency of occurrence of events rated on 4-point scale, where higher scores indicate more frequent occurrence and higher frequency of communication about sex | Miller KS, Kotchick BA, Dorsey S, Forehand R, Ham AY. Family communication about sex: what are parents saying and are their adolescents listening?. *Fam Plann Perspect*. 1998;30(5):218-235. |
|  | CoRH | Celebration of racial heritage subscale, Racial Socialization Scale | Murry et al., 2019a^7^ | Assesses positive racial socialization pracitices | Cronbach a > .84 at pre- and post-tests. Racial Socialization Scale valid and reliable. Subscale used in study probably adaptation of original scale, including selective use of items and use of different scales (3-point vs. 5-point). | 5 | Questionnaire administered through computer-assisted interview | Frequency of occurrence of events rated on 3-point scale, where higher scores indicate more frequent occurrence and higher levels of positive racial socialization | Hughes D, Johnson D. Correlates in Children's Experiences of Parents' Racial Socialization Behaviors. J*ournal of Marriage and Family.* 2001;63(4):981-995. doi:https://doi.org/10.1111/j.1741-3737.2001.00981.x |
|  | SIQS | Substance Intention Questions Scale | Murry et al., 2019a^7^ | Assesses intent to engage in risk behaviour | Cronbach a > .86 at pre- and post-tests. Scale cannot be identified in referenced study, as the referenced study explicitly measures expectations and willingness, rather than intentions. However, the examples provided in the present study include 1 item which probably measures behavioural intentions and 1 item which probably measures behavioural expectations. | 8 | Questionnaire administered through computer-assisted interview | Agreement with statements/likelihood of occurrence of events rated in 4-point scale, where higher scores indicate higher levels of agreement or likelihood and greater intent to engage in risk behaviour. | Gibbons FX, Gerrard M, Blanton H, Russell DW. Reasoned action and social reaction: willingness and intention as independent predictors of health risk. *J Pers Soc Psychol*. 1998;74(5):1164-1180. doi:10.1037//0022-3514.74.5.1164 |
|  | AwDPS | Affiliation with Deviant Peers Scale | Murry et al., 2019a^7^ | Assesses affiliation with peers engaging in externalizing behaviours, substance use, and sexual risk behaviours | Cronbacch’s a > .88 for pre- and post-tests. Scale cannot be identified in referenced book. It most probably refers to the Exposure to Delinquent Peers Scale (cf. p. 90), which was seemingly adapted (5-point scoring system in referenced book vs. 4-point scale in present study) | 14 items for Exposure to Delinquent Peers Scale, no information number of items used in present study | Questionnaire administered through computer-assisted interview | Number of close friends who engaged in externalizing behaviours, substance use, and sexual risk behaviours rated on 4-point scale, where higher scores indicate higher numbers of friends engaging in these behaviours | Elliot, DS, Huizinga, D, Ageton, S. Explaining delinquency and drug use. Beverly Hills: Sage; 1985 |
|  | SRS | Sexual Risk Survey | Murry et al., 2019a^7^ | Assesses vaginal, anal, and oral sexual behaviours | Unclear reliability and validity | 37 | Questionnaire administered through computer-assisted interview | Dichotomous scales for yes/no questions and count scales for questions about frequency. Higher scores indicate greater engagement in risky behaviours. | Jemmott JB 3rd, Jemmott LS, Fong GT, McCaffree K. Reducing HIV risk-associated sexual behavior among African American adolescents: testing the generality of intervention effects. *Am J Community Psychol*. 1999;27(2):161-187. doi:10.1007/BF02503158 |
|  | MFS | Monitoring the Future Scale | Murry et al., 2019a^7^ | Assesses use of cigarettes, alcohol, marijuana, cocaine, hallucinogens, methamphetamines, heroin, huffing, ecstasy, or prescription drugs | Referenced Monitoring the Future Scale is reliable and valid. The 28 items are seemingly a subset of items included in the referenced scale. No information on reliability and validity of subset. | 28 | Questionnaire administered through computer-assisted interview | Frequency of occurrence of use of different substances, probably rated on 7-point scale, where higher scores indicate greater engagement in substance use behaviours | Johnston, L D, Bachman, J G, O’Malley, P M. Monitoring the future: Questionnaire responses from the nation’s high school seniors. Ann Arbor: University of Michigan, Survey Research Center; 1993 |
|  | Y-AN/EaRE | Articulated Norms and Expectations about Risk Engagement, youth report | Murry et al., 2019a^7^ | Assesses adolescent’s perceptions of rules pertaining to alcohol, drugs, and sex | Unclear, not unambiguously identifiable from provided reference | 9 | Questionnaire administered through computer-assisted interview | Level of engagement in conversations about rules rated on 5-point scale where higher scores indicate higher levels of engagement. | Spoth R, Redmond C, Shin C. Direct and indirect latent-variable parenting outcomes of two universal family-focused preventive interventions: extending a public health-oriented research base. *J Consult Clin Psychol*. 1998;66(2):385-399. doi:10.1037//0022-006x.66.2.385 |
|  | P-AN/EaRE | Articulated Norms and Expectations about Risk Engagement, parent report | Murry et al., 2019b^2^ | Assesses adolescent’s perceptions of rules pertaining to alcohol, drugs, and sex | Unclear, not unambiguously identifiable from provided reference | 9 | Questionnaire administered through computer-assisted interview | Level of engagement in conversations about rules rated on 5-point scale where higher scores indicate higher levels of engagement. | Spoth R, Redmond C, Shin C. Direct and indirect latent-variable parenting outcomes of two universal family-focused preventive interventions: extending a public health-oriented research base. *J Consult Clin Psychol*. 1998;66(2):385-399. doi:10.1037//0022-006x.66.2.385  Format: |
|  | EDSIS | Eating Disorder Symptom Impact Scale | Truttmann et al., 2020^4^ | Assesses specific caregiving difficulties  for families of people with EDs | Valid and reliable | 24 | Self-report | Caregiving difficulties rated on 5 point scale, where higher scores indicate more  diculties. | Sepulveda, A.R.; Whitney, J.; Hankins, M.; Treasure, J. Development and validation of an Eating Disorders  Symptom Impact Scale (EDSIS) for carers of people with eating disorders. Health Qual. Life Outcomes 2008, 6,  28. |
|  | SCL-90-R | Symptom Checklist Global Severity Index | Truttmann et al., 2020^4^ | Assesses a broad range of psychopathological symptoms of caregivers | Valid and reliable | 90 | Self -eport | Severity of symptoms rated on 5-point scale, where higher scores indicate higher levels of psychopathology | Franke, G.H. Die Symptom-Checkliste von Derogatis (SCL-90-R)—Deutsche; Version 2; Beltz: Göttingen,  Germany, 2002. |
|  | BDI-II | Beck Depression Inventory | Truttmann et al., 2020^4^ | Assesses symptoms of depression of caregivers | Valid and reliable | 21 | Self-report | Symptoms of depression rated on 4-point scale, where higher scores  indicate higher levels of depression. | Hautzinger, M.; Keller, F.; Kühner, C. Beck Depressions Inventar, 2. Auflage (BDI-II); Pearson: Hallbergmoos,  Germany, 2006.  ^41^Kühner, C.; Bürger, C.; Keller, F.; Hautzinger, M. Reliabilität und Validität des revidierten  Beck-Depressionsinventars (BDI-II). Nervenarzt 2007, 78, 651–656. |
|  | STAI | State and Trait Anxiety Inventory | Truttmann et al., 2020^4^ | Assesses two kinds of anxiety: “State-anxiety” ( characterised as inner tension and concerns  toward future events in caregivers that vary across time and situations) and “trait anxiety” (the tendency  of experiencing fear in general) | Valid and reliable | 40 | Self-report | Anxiety rated on 4-point scale, where  higher scores indicate higher levels of anxiety. | Laux, L.; Glanzmann, P.; Scha ner, P.; Spielsberger, C.D. STAI—Das State-Trait-Angstinventar, 1st ed.; Beltz:  Weinheim, Germany, 1981. |
|  | CASK Total | Caregiver Skills scale | Truttmann et al., 2020^4^ | Assesses skills in caregivers | Valid and reliable | 27 | Self-report | Skill levels rated on visual analogue scale (0–100), where higher scores indicate  higher skill levels. | Hibbs, R.; Rhind, C.; Salerno, L.; Lo Coco, G.; Goddard, E.; Schmidt, U.; Micali, N.; Gowers, S.; Beecham, J.;  Macdonald, P.; et al. Development and validation of a scale to measure caregiver skills in eating disorders.  Int. J. Eat. Disord. 2015, 48, 290–297. |
|  | CES-D | Center for Epidemiological Studies Depression Scale | Wade et al., 2019a^8^ | Assesses depressive symptoms of caregivers over past week | Valid and reliable | 20 | Self-report | Total score range from 0-60, where higher scores indicate higher depression; scores of 16 and above indicate clinically significant levels of depression | Radloff LS. The CES-D scale: a self-report depression scale for  research in the general population. Appl Psychol Meas. 1977;1(3):  385–401. |
|  | BSI | Brief Symptom Inventory | Wade et al., 2019a^8^ | Assesses broad range of psychiatric symptoms of caregivers over the past week | Valid and reliable | 53 | Self-report | Normative comparison with T score with mean of 50 and SD of 10. Higher scores indicate more psychiatric distress, scores of 63 clinically significant. | Derogatis LR. The Brief Symptom Inventory (BSI) Administration,  Scoring, and Procedures Manual. Minneapolis, MN: National Computer  Systems; 1993. |
|  | PedsQL, parent report | Pediatric Quality of Life Inventory, caregiver report | Wade et al., 2019b^9^ | Assesses quality of life of youth by caregiver | Valid and reliable | 23 | Self-report | Higher scores indicate better QoL (normative sample: 82.7 [SD 15.4]) | Varni, J. W., Burwinkle, T. M., Seid, M., & Skarr, D. (2003). The PedsQL  4.0 as a pediatric population health measure: Feasibility, reliability, and  validity. Ambulatory Pediatrics, 3, 329–341. http://dx.doi.org/10.1367/  1539-4409(2003)0030329:TPAAPP2.0.CO;2 |
|  | PedsQL, parent report | Pediatric Quality of Life Inventory, adolescent report | Wade et al., 2019b^9^ | Assesses quality of life of youth | Valid and reliable | 23 | Self-report | Higher scores indicate better QoL (normative sample: 83.8 [SD 12.6]) | Varni, J. W., Seid, M., & Rode, C. A. (1999). The PedsQL: Measurement  model for the pediatric quality of life inventory. Medical Care, 37,  126–139. http://dx.doi.org/10.1097/00005650-199902000-00003 |
|  | HBI cognitive, parent report | Health and Behaviour Inventory, caregiver report | Wade et al., 2019b^9^ | Assesses cognitive symptoms | Valid and reliable | 50 | Self-report | Cognitive symptoms rated on 4-point scale, where higher scores indicate more TBI symptoms | Yeates, K. O., Luria, J., Bartkowski, H., Rusin, J., Martin, L., & Bigler,  E. D. (1999). Postconcussive symptoms in children with mild closed  head injuries. The Journal of Head Trauma Rehabilitation, 14, 337–350.  http://dx.doi.org/10.1097/00001199-199908000-00003 |
|  | HBI somatic, parent report | Health and Behaviour Inventory, caregiver report | Wade et al., 2019b^9^ | Assesses somatic symptoms | Valid and reliable | 50 | Self-report | Somatic symptoms rated on 4-point scale, where higher scores indicate more TBI symptoms | Yeates, K. O., & Taylor, H. G. (2005). Neurobehavioural outcomes of mild  head injury in children and adolescents. Pediatric Rehabilitation, 8,  5–16. http://dx.doi.org/10.1080/13638490400011199 |
|  | HBI cognitive, youth report | Health and Behaviour Inventory, adolescent report | Wade et al., 2019b^9^ | Assesses cognitive symptoms | Valid and reliable | 50 | Self-report | Cognitive symptoms rated on 4-point scale, where higher scores indicate more TBI symptoms | Yeates, K. O., Luria, J., Bartkowski, H., Rusin, J., Martin, L., & Bigler,  E. D. (1999). Postconcussive symptoms in children with mild closed  head injuries. The Journal of Head Trauma Rehabilitation, 14, 337–350.  <http://dx.doi.org/10.1097/00001199-199908000-00003>  Yeates, K. O., & Taylor, H. G. (2005). Neurobehavioural outcomes of mild  head injury in children and adolescents. Pediatric Rehabilitation, 8,  5–16. http://dx.doi.org/10.1080/13638490400011199 |
|  | HBI somatic, youth report | Health and Behaviour Inventory, adolescent report | Wade et al., 2019b^9^ | Assesses somatic symptoms | Valid and reliable | 50 | Self-report | Somatic symptoms rated on 4-point scale, where higher scores indicate more TBI symptoms | Yeates, K. O., Luria, J., Bartkowski, H., Rusin, J., Martin, L., & Bigler,  E. D. (1999). Postconcussive symptoms in children with mild closed  head injuries. The Journal of Head Trauma Rehabilitation, 14, 337–350.  <http://dx.doi.org/10.1097/00001199-199908000-00003>  Yeates, K. O., & Taylor, H. G. (2005). Neurobehavioural outcomes of mild  head injury in children and adolescents. Pediatric Rehabilitation, 8,  5–16. <http://dx.doi.org/10.1080/13638490400011199> |
| Further | Y-WAI | Working Alliance Inventory (Client), youth report | Freeman et al., 2013^10^ | Assesses the relationship between clinician and client (working alliance) globally as well as on 3 subscales | Valid and reliable | 36 | No information, but likely self-report | Frequency of occurrence of events rated on 7-point scale, where higher scores indicate more frequent occurrence and a more positive working alliance | ^50^Horvath AO, Greenberg LS. Development and validation of the working alliance inventory. *J Counsel Psychol.* 1989;36:223-33 |
|  | P-WAI | Working Alliance Inventory (Client), parent report | Freeman et al., 2013^10^ | Assesses the relationship between clinician and client globally as well as on 3 subscales | Valid and reliable | 36 | No information, but likely self-report | Frequency of occurrence of events rated on 7-point scale, where higher scores indicate more frequent occurrence and a more positive working alliance | Horvath AO, Greenberg LS. Development and validation of the working alliance inventory. *J Counsel Psychol.* 1989;36:223-33 |
|  | P-PE | Program Evaluation, parent report | Wade et al., 2019c^11^ | Assesses participant evaluation  of their assigned intervention modality | In-house/ custom | PE, PB, and OS combined: 19 | Self-report | 5-point Likert scale to rank modality convenience and benefit. These scores were summed and modality was ranked from most preferred to least preferred. Ranking was compared with actual assignment and coded as “Yes” if matching or “No” when not matching. | Wade D, Als N, Bell V, et al. Providing psychological support to people in intensive care: development and feasibility study of a nurse-led intervention to prevent acute stress and long-term morbidity. *BMJ Open*. 2018;8(7):e021083. Published 2018 Jul 23. doi:10.1136/bmjopen-2017-021083 |
|  | Y-PE | Program Evaluation, adolescent report | Wade et al., 2019c^11^ | Assesses participant evaluation  of their assigned intervention modality | In-house/ custom | PE, PB, and OS combined: 19 | Self-report | 5-point Likert scale to rank modality convenience and benefit. These scores were summed and modality was ranked from most preferred to least preferred. Ranking was compared with actual assignment and coded as “Yes” if matching or “No” when not matching. | Wade D, Als N, Bell V, et al. Providing psychological support to people in intensive care: development and feasibility study of a nurse-led intervention to prevent acute stress and long-term morbidity. *BMJ Open*. 2018;8(7):e021083. Published 2018 Jul 23. doi:10.1136/bmjopen-2017-021083 |
|  | P-PB | Program Benefit, caregiver report | Wade et al., 2019c^11^ | Assesses the perceived benefit  of the assigned intervention  modality | In-house/ custom | PE, PB, and OS combined: 19 | Self-report | 5-point Likert scale to rank modality convenience and benefit. These scores were summed and modality was ranked from most preferred to least preferred. Ranking was compared with actual assignment and coded as “Yes” if matching or “No” when not matching. | Wade D, Als N, Bell V, et al. Providing psychological support to people in intensive care: development and feasibility study of a nurse-led intervention to prevent acute stress and long-term morbidity. *BMJ Open*. 2018;8(7):e021083. Published 2018 Jul 23. doi:10.1136/bmjopen-2017-021083 |
|  | Y-PB | Program Benefit, adolescent report | Wade et al., 2019c^11^ | Assesses the perceived benefit  of the assigned intervention  modality | In-house/ custom | PE, PB, and OS combined: 19 | Self-report | 5-point Likert scale to rank modality convenience and benefit. These scores were summed and modality was ranked from most preferred to least preferred. Ranking was compared with actual assignment and coded as “Yes” if matching or “No” when not matching. | Wade D, Als N, Bell V, et al. Providing psychological support to people in intensive care: development and feasibility study of a nurse-led intervention to prevent acute stress and long-term morbidity. *BMJ Open*. 2018;8(7):e021083. Published 2018 Jul 23. doi:10.1136/bmjopen-2017-021083 |
|  | P-OS | Satisfaction survey, caregiver report | Wade et al., 2019c^11^ | Assesses perceived overall satisfaction with the assigned intervention | Valid and reliable | PE, PB, and OS combined: 19 | Self-report | Satisfaction rated on 10-point scale, where higher scores indicate higher satisfaction | Wade, S. L., Walz, N. C., Carey, J., McMullen, K. M., Cass,  J., Mark, E., & Yeates, K. O. (2011). Effect on behavior  problems of teen online problem-solving for adolescent  traumatic brain injury. Pediatrics, 128, e947–e953. |
|  | Y-OS | Satisfaction survey, adolescent report | Wade et al., 2019c^11^ | Assesses perceived overall satisfaction with the assigned intervention | Valid and reliable | PE, PB, and OS combined: 19 | Self-report | Satisfaction rated on 10-point scale, where higher scores indicate higher satisfaction | Wade, S. L., Walz, N. C., Carey, J., McMullen, K. M., Cass,  J., Mark, E., & Yeates, K. O. (2011). Effect on behavior  problems of teen online problem-solving for adolescent  traumatic brain injury. Pediatrics, 128, e947–e953. |

**Notes**: References from publications included in systematic review. More appropriate references than those in the original studies were provided only where necessary and appropriate. Where no abbreviations/acronyms where available, custom abbreviations were used.

# **References**

1. Harris MA, Freeman KA, Duke DC. Seeing Is Believing: Using Skype to Improve Diabetes Outcomes in Youth. *Diabetes Care*. Aug 2015;38(8):1427-34. doi:10.2337/dc14-2469

2. Murry VM, Kettrey HH, Berkel C, Inniss-Thompson MN. The Pathways for African American Success: Does Delivery Platform Matter in the Prevention of HIV Risk Vulnerability Among Youth? *J Adolesc Health*. Aug 2019b;65(2):255-261. doi:10.1016/j.jadohealth.2019.02.013

3. Kurowski BG, Taylor HG, McNally KA, et al. Online Family Problem-Solving Therapy (F-PST) for Executive and Behavioral Dysfunction After Traumatic Brain Injury in Adolescents: A Randomized, Multicenter, Comparative Effectiveness Clinical Trial. *J Head Trauma Rehabil*. May/Jun 2020;35(3):165-174. doi:10.1097/htr.0000000000000545

4. Truttmann S, Philipp J, Zeiler M, et al. Long-Term Efficacy of the Workshop Vs. Online SUCCEAT (Supporting Carers of Children and Adolescents with Eating Disorders) Intervention for Parents: A Quasi-Randomised Feasibility Trial. *J Clin Med*. Jun 18 2020;9(6)doi:10.3390/jcm9061912

5. Duke DC, Wagner DV, Ulrich J, Freeman KA, Harris MA. Videoconferencing for Teens With Diabetes: Family Matters. *J Diabetes Sci Technol*. Jul 2016;10(4):816-23. doi:10.1177/1932296816642577

6. Riley AR, Duke DC, Freeman KA, Hood KK, Harris MA. Depressive Symptoms in a Trial Behavioral Family Systems Therapy for Diabetes: A Post Hoc Analysis of Change. *Diabetes Care*. Aug 2015;38(8):1435-40. doi:10.2337/dc14-2519

7. Murry VM, Berkel C, Inniss-Thompson MN, Debreaux ML. Pathways for African American Success: Results of Three-Arm Randomized Trial to Test the Effects of Technology-Based Delivery for Rural African American Families. *J Pediatr Psychol*. Apr 1 2019a;44(3):375-387. doi:10.1093/jpepsy/jsz001

8. Wade SL, Cassedy AE, McNally KA, et al. A Randomized Comparative Effectiveness Trial of Family-Problem-Solving Treatment for Adolescent Brain Injury: Parent Outcomes From the Coping with Head Injury through Problem Solving (CHIPS) Study. *J Head Trauma Rehabil*. Nov/Dec 2019a;34(6):E1-e9. doi:10.1097/htr.0000000000000487

9. Wade SL, Cassedy AE, Sklut M, et al. The Relationship of Adolescent and Parent Preferences for Treatment Modality With Satisfaction, Attrition, Adherence, and Efficacy: The Coping With Head Injury Through Problem-Solving (CHIPS) Study. *J Pediatr Psychol*. Apr 1 2019b;44(3):388-401. doi:10.1093/jpepsy/jsy087

10.Freeman KA, Duke DC, Harris MA. Behavioral health care for adolescents with poorly controlled diabetes via Skype: does working alliance remain intact? *J Diabetes Sci Technol*. May 1 2013;7(3):727-35. doi:10.1177/193229681300700318

11.Wade SL, Cassedy AE, Taylor HG, et al. Adolescent quality of life following family problem-solving treatment for brain injury. *J Consult Clin Psychol*. Nov 2019c;87(11):1043-1055. doi:10.1037/ccp0000440
