# Supplementary material for: Comparing Digital Versus Face-to-Face Delivery of Systemic Psychotherapy Interventions: Systematic Review and Meta-Analysis of Randomized Controlled Trials
Source: Interact J Med Res. 2025 Feb 24;14:e46441. doi: 10.2196/46441 (PMC11894358; doi:10.2196/46441)
Supplement: Multimedia Appendix 9 [file ijmr_v14i1e46441_app9.docx]

**Multimedia Appendix 9:** Comparison Tables

Comparison of Youth Outcomes for Therapist-guided Digital versus Face-to-face Delivery Modalities

For the 11 youth outcomes, we calculated MDs and CIs for outcome measures as compared for therapist-guided digital and face-to-face delivery modalities of 2 trials (BFST-D and F-PST trials) (Table 2). One post-test outcome measure, the PedsQL in the F-PST trial, and 1 follow-up outcome measure, the youth-reported HBI somatic symptoms in the F-PST trial, was significantly higher for therapist-guided digital compared to face-to-face, indicating higher parent-reported quality of life for youth in the therapist-guided digital condition and significantly higher youth-reported somatic symptoms in the face-to-face delivery condition compared to the therapist-guided digital condition. There was no evidence for other statistically significant differences found between delivery conditions, all other CIs of MDs fell within and exceeded the minimal important difference.

**Table 2.** Means and mean differences of youth outcomes for face-to-face and therapist-guided digital delivery conditions at post-test and follow-up (N=449^a^).

|  |  |  |  | Face-to-face delivery | | Digital delivery | | Mean difference (95% CI) |
| --- | --- | --- | --- | --- | --- | --- | --- | --- |
| Post-test/ Follow-up | Trial | Publication | Outcome | n | Mean (SD) | n | Mean (SD) |  |
|  |  |  |  |  |  |  |  |  |
| Post-test | BFST-D | Harris et al (2015)^1^ | HbA1c | 35 | 10.45 (2.05) | 31 | 10.40 (1.66) | 0.05  (-0.85 to 0.95) |
|  |  |  | Y-DSMP | 35 | 58.07 (8.25) | 31 | 56.10 (13.30) | 1.97  (-3.45 to 7.39) |
|  | F-PST | Kurowski et al (2020)^2^ | BRIEF, parent report | 22 | 65.1 (12.5) | 43 | 59.9 (14.40) | 5.20 (-1.57 to 11.97) |
|  |  |  | BRIEF, youth report | 22 | 57.8 (13.72) | 43 | 54.1 (15.08) | 3.70 (-3.59 to 10.99) |
|  |  |  | SDQ | 22 | 14.1 (5.88) | 43 | 11.7 (6.86) | 2.40 (-0.80 to 5.60) |
|  |  | Wade et al (2019b)^3^ | PedsQL, parent report | 22 | 62 (15.71) | 43 | 70.9 (18.49) | -8.90 (-17.48 to -0.32) |
|  |  |  | PedsQL, youth report | 22 | 69.2 (15.67) | 43 | 72.2 (18.23) | -3.00 (-11.57 to  5.57) |
|  |  |  | HBI cognitive, parent report | 22 | 19.6 (8.16) | 43 | 17.2 (9.31) | 2.40 (-2.00 to 6.80) |
|  |  |  | HBI somatic, parent report | 22 | 7.0 (4.60) | 43 | 6.1 (5.38) | 0.90 (-1.61 to 3.41) |
|  |  |  | HBI cognitive, youth report | 22 | 18.1 (7.41) | 43 | 15.8 (8.66) | 2.30 (-1.74 to 6.34) |
|  |  |  | HBI somatic, youth report | 22 | 7.7 (5.53) | 43 | 7.1 (6.36) | 0.60 (-2.39 to 3.59) |
| Follow-up | BFST-D | Harris et al (2015)^1^ | HbA1c | 35 | 10.31 (1.95) | 29 | 10.61 (1.83) | -0.30  (-1.23 to 0.63) |
|  |  |  | Y-DSMP | 35 | 57.66 (9.50) | 29 | 55.55 (12.81) | 2.11  (-3.52 to 7.74) |
|  |  | Kurowski et al (2020)^2^ | BRIEF, parent report | 22 | 61.3 (12.5) | 44 | 60.0 (14.40) | 1.30 (-5.44 to 8.04) |
|  |  |  | BRIEF, youth report | 22 | 58.2 (13.72) | 44 | 52.5 (15.77) | 5.70 (-1.69 to 13.09) |
|  |  |  | SDQ | 22 | 13.1 (6.37) | 44 | 11.6 (7.54) | 1.50 (-1.97 to 4.97) |
|  | F-PST | Wade et al (2019b)^3^ | PedsQL, parent report | 22 | 67 (16.18) | 44 | 71.3 (19.04) | -4.30 (-13.10 to 4.50) |
|  |  |  | PedsQL, parent report | 22 | 69.3 (15.57) | 44 | 74.7 (18.37) | -5.40 (-13.87 to 3.07) |
|  |  |  | HBI cognitive, parent report | 22 | 19.1 (8.58) | 44 | 16.0 (9.95) | 3.10 (-1.54 to 7.74) |
|  |  |  | HBI somatic, parent report | 22 | 6.2 (4.27) | 44 | 5.5 (5.17) | 0.70 (-1.65 to 3.05) |
|  |  |  | HBI cognitive, youth report | 22 | 18.1 (7.60) | 44 | 14.8 (8.95) | 3.30 (-0.83 to 7.43) |
|  |  |  | HBI somatic, youth report | 22 | 9.8 (4.92) | 44 | 6.6 (5.90) | 3.20 (0.50 - 5.90) |

^a^ Sum of n at trial level

^b^ Self-guided digital delivery intervention

Abbreviations: HbA1c, hemoglobin A1c; Y-DSMP, Diabetes Self-Management Profile, youth report; Y-REI, Youth Risk Engagement Intentions; AN/EaREP, Articulated Norms and Expectations about Risk Engagement; BRIEF, Behaviour Rating Inventory of Executive Function, parent-report; SDQ, Strengths and Difficulties Questionnaire; PedsQL, Pediatric Quality of Life Inventory; HBI, Health and Behaviour Inventory

Comparison of Parent Outcomes for Therapist-guided Digital versus Face-to-face Delivery Modalities

For the 10 parent outcomes, we calculated MDs and CIs for outcome measures for therapist-guided digital and face-to-face delivery modalities of 3 trials (BFST-D, F-PST, and SUCCEAT trials) (Table 3). For post-test, the results for the CES-D and BSI were significantly higher in the face-to-face condition, indicating higher levels of depression and psychiatric symptoms, as compared to the therapist-guided digital delivery condition. The CI for SCL-90-R in the SUCCEAT trial remained within the range defined for the minimal important difference, indicating equivalence across a broad range of psychopathological symptoms at post-test between the digital and face-to-face conditions. For follow-up measures, the BSI results were significantly higher for the therapist-guided digital condition, showing higher levels of psychiatric symptoms in the therapist-guided digital condition compared to the face-to-face delivery condition. There was no evidence for other statistically significant differences found, all other CIs of MDs fell within and exceeded the minimal important difference.

**Table 3.** Means and mean differences of parent outcomes for face-to-face and therapist-guided digital delivery conditions at post-test and follow-up (N=226^a^).

|  |  |  |  | Face-to-face delivery | | Digital delivery | | Mean difference (95% CI) |
| --- | --- | --- | --- | --- | --- | --- | --- | --- |
| Post-test/ Follow-up | Trial | Publication | Outcome | N | Mean (SD) | n | Mean (SD) |  |
|  |  |  |  |  |  |  |  |  |
| Post-test | BFST-D | Harris et al (2015)^1^ | P-DSMP | 35 | 53.61 (11.83) | 31 | 52.84 (13.49) | 0.77  (-5.39 to 6.93) |
|  | F-PST | Wade et al (2019a)^4^ | CES-D | 22 | 15.1 (1.81) | 43 | 12.8 (1.53) | 2.30 (1.42- 3.18) |
|  |  |  | BSI | 22 | 56.1 (2.46) | 43 | 53.3 (2.02) | 2.80 (1.61- 3.99) |
|  | SUCCEAT | Truttmann et al (2020)^5^ | GHQ | 48 | 1.84 (2.74) | 46 | 2.14 (2.92) | -0.30 (-1.45 to 0.85) |
|  |  |  | EDSIS | 48 | 22.59 (13.24) | 46 | 19.83 (11.82) | 2.76 (-2.31 to 7.83) |
|  |  |  | SCL-90-R | 48 | 0.24 (0.26) | 46 | 0.31 (0.36) | -0.07 (-0.20 to 0.06) |
|  |  |  | BDI-II | 48 | 6.45 (5.43) | 46 | 7.72 (8.18) | -1.27 (-4.09 to 1.55) |
|  |  |  | STAI Trait | 48 | 37.86 (8.62) | 46 | 38.01 (10.57) | -0.15 (-4.06 to 3.76) |
|  |  |  | STAI State | 48 | 39.80 (10.16) | 46 | 41.84 (12.17) | -2.04 (-6.58 to 2.50) |
|  |  |  | CASK Total | 48 | 75.33 (13.68) | 46 | 74.08 (12.63) | 1.25 (-4.07 to 6.57) |
| Follow-up | BFST-D | Harris et al (2015)^1^ | P-DSMP | 35 | 51.83 (11.88) | 29 | 50.09 (12.91) | 1.74  (-4.39 to 7.87) |
|  | F-PST | Wade et al (2019a)^4^ | CES-D | 22 | 11.8 (2.03) | 44 | 11.4 (1.65) | 0.40 (-0.58 to 1.38) |
|  |  |  | BSI | 22 | 51.7 (2.42) | 44 | 53.3 (2.02) | -1.60 (-2.77 to -0.43) |
|  | SUCCEAT | Truttmann et al (2020)^5^ | GHQ | 44 | 1.94 (1.39) | 36 | 1.39 (2.18) | 0.55 (-0.27 to 1.37) |
|  |  |  | EDSIS | 44 | 20.28 (16.29) | 46 | 16.60 (11.41) | 3.68 (-2.41 to 9.77) |
|  |  |  | SCL-90-R | 44 | 0.33 (0.37) | 46 | 0.26 (0.26) | 0.07 (-0.07 to 0.21) |
|  |  |  | BDI-II | 44 | 6.80 (6.24) | 46 | 5.74 (5.97) | 1.06 (-1.62 to 3.74) |
|  |  |  | STAI Trait | 44 | 36.16 (9.65) | 46 | 36.56 (9.50) | -0.40 (-4.61 to 3.81) |
|  |  |  | STAI State | 44 | 37.85 (10.31) | 46 | 37.99 (10.31) | -0.14 (-4.40 to 4.12) |
|  |  |  | CASK Total | 44 | 79.53 (15.25) | 46 | 77.01 (12.87) | 2.52 (-3.64 to 8.68) |

^a^ Sum of n at trial level

Abbreviations: P-DSMP, Diabetes Self-Management Profile, parent report; CES-D, Center for Epidemiological Studies Depression Scale; BSI, Brief Symptom Inventory; GHQ, General Health Questionnaire; EDSIS, Eating Disorder Symptom Impact Scale; SCL, Symptom Checklist Global Severity Index; BDI, Beck Depression Inventory; STAI, State and Trait Anxiety Inventory; CASK, Caregiver Skills Scale.

Comparison of Youth Outcomes for Self-guided Digital versus Face-to-face Delivery Modalities

We calculated MDs and CIs for 10 youth outcome measures for self-guided digital and face-to-face delivery modalities of 2 trials (F-PST, PAAS) (Table 4). The Y-REI in the PAAS trial was significantly higher in the face-to-face condition compared to the self-guided digital delivery condition, indicating higher levels of risk engagement intentions in youth for the face-to-face condition. The PedsQL in the F-PST trial was significantly higher for the self-guided digital delivery condition, indicating higher quality of life compared to the face-to-face condition. At follow-up, 4 youth outcomes were significantly different in the F-PST trial comparing self-guided digital and face-to-face delivery modalities. The PedsQL showed higher parent-rated quality of life for youth in the self-guided digital delivery condition compared to the face-to-face condition. Results for parent-rated cognitive and somatic youth symptoms (HBI cognitive and HBI somatic) and youth-rated somatic symptoms (HBI somatic) indicated higher parent-rated cognitive and somatic health behavior symptoms for youth in the face-to-face condition compared to the self-guided digital delivery condition. There was no evidence for other statistically significant differences found, all other CIs of MDs fell within and exceeded the minimal important difference.

**Table 4.** Means and mean differences of youth outcomes for face-to-face and self-guided digital delivery conditions at post-test and follow-up (N=297^a^)

|  |  |  |  | Face-to-face delivery | | Digital delivery | |  |
| --- | --- | --- | --- | --- | --- | --- | --- | --- |
| Post-test/ Follow-up | Trial | Publication | Outcome | n | Mean (SD) | n | Mean (SD) | Mean difference (95% CI) |
|  |  |  |  |  |  |  |  |  |
| Post-test | PAAS | Murry et al (2019b)^6^ | Y-REI | 100 | 9.29 (3.06) | 124 | 8.47 (1.31) | 0.82 (0.18 to 1.46) |
|  | F-PST | Kurowski (2020)^2^ | BRIEF, parent report | 22 | 65.1 (12.5) | 51 | 59.5 (14.56) | 5.60 (-0.98 to 12.18) |
|  |  |  | BRIEF, youth report | 22 | 57.8 (13.72) | 51 | 54.3 (15.29) | 3.50 (-3.60 to 10.60) |
|  |  |  | SDQ | 22 | 14.1 (5.88) | 51 | 12.6 (7.28) | 1.50 (-1.67 to 4.67) |
|  |  | Wade et al (2019b)^3^ | PedsQL, parent report | 22 | 62 (15.71) | 51 | 71.7 (18.85) | -9.70 (-18.06 to -1.34) |
|  |  |  | PedsQL, youth report | 22 | 69.2 (15.67) | 51 | 73.1 (18.49) | -3.90 (-12.18 to 4.38) |
|  |  |  | HBI cognitive, parent report | 22 | 19.6 (8.16) | 51 | 15.4 (9.49) | 4.20 (-0.09 to 8.49) |
|  |  |  | HBI somatic, parent report | 22 | 7.0 (4.60) | 51 | 4.6 (5.43) | 2.40 (-0.03 to 4.83) |
|  |  |  | HBI cognitive, youth report | 22 | 18.1 (7.41) | 51 | 16.4 (8.71) | 1.70 (-2.21 to 5.61) |
|  |  |  | HBI somatic, youth report | 22 | 7.7 (5.53) | 51 | 7.2 (6.36) | 0.50 (-2.40 to 3.40) |
| Follow-Up | F-PST | Kurowski (2020)^2^ | BRIEF, parent report | 22 | 61.3 (12.5) | 48 | 57.0 (14.56) | 4.30 (-2.35 to 10.9) |
|  |  |  | BRIEF, youth report | 22 | 58.2 (13.72) | 48 | 55.8 (16.02) | 2.40 (-4.91 to 9.71) |
|  |  |  | SDQ | 22 | 13.1 (6.37) | 48 | 10.8 (7.28) | 2.30 (-1.07 to 5.67) |
|  |  | Wade et al (2019b)^3^ | PedsQL, parent report | 22 | 67 (16.18) | 48 | 76.6 (18.98) | -9.60 (-18.23 to -0.97) |
|  |  |  | PedsQL, youth report | 22 | 69.3 (15.57) | 48 | 74.8 (18.15) | -5.50 (-13.79 to 2.79) |
|  |  |  | HBI cognitive, parent report | 22 | 19.1 (8.58) | 48 | 12.3 (9.91) | 6.80 (2.25 - 11.35) |
|  |  |  | HBI somatic, parent report | 22 | 6.2 (4.27) | 48 | 3.8 (5.12) | 2.40 (0.10 - 4.70) |
|  |  |  | HBI cognitive, youth report | 22 | 18.1 (7.60) | 48 | 15.9 (8.31) | 2.20 (-1.75 to 6.15) |
|  |  |  | HBI somatic, youth report | 22 | 9.8 (4.92) | 48 | 6.8 (5.89) | 3.00 (0.35 - 5.65) |

a: Sum of n at trial level

Abbreviations: BRIEF, Behaviour Rating Inventory of Executive Function; SDQ, Strengths and Difficulties Questionnaire; Y-REI, Youth Risk Engagement Intentions; PedsQL, Pediatric Quality of Life Inventory; HBI, Health and Behaviour Inventory

Comparison of Parent Outcomes for Self-guided digital versus Face-to-face Delivery Modalities

A total of 2 measures across 1 publication reporting on 1 trial (F-PST trial^39^) was used for parent-related outcomes at both post-test and follow-up time points (see Multimedia Appendix 4 for measures used).

We calculated MDs and CIs for outcome measures for self-guided digital and face-to-face delivery modalities of 1 trial (F-PST) (Table 5). The two post-test measures in the F-PST trial^39^ were significantly higher for the face-to-face condition, namely the CES-D and the BSI, indicating higher levels of depression and psychiatric symptoms at post-test for the face-to-face condition compared to the digital delivery condition. One follow-up measure in the F-PST trial^39^ was significantly higher in the self-guided digital delivery condition, namely CES-D, indicating higher levels of parental depression when compared to the face-to-face delivery condition. No other significant differences were found, all other CIs of MDs fell within and exceeded the minimal important difference.

**Table 5.** Means and mean differences of parent outcomes for face-to-face and self-guided digital delivery conditions at post-test and follow-up (N=73^a^).

|  |  |  |  | Face-to-face delivery | | Digital delivery | |  |
| --- | --- | --- | --- | --- | --- | --- | --- | --- |
| Post-test/ Follow-up | Trial | Publication | Outcome | n | Mean (SD) | n | Mean (SD) | Mean difference (95% CI) |
|  |  |  |  |  |  |  |  |  |
| Post-test | F-PST | Wade et al (2019a)^4^ | CES-D | 22 | 15.1 (1.81) | 51 | 13.5 (1.42) | 1.60 (0.75 - 2.45) |
|  |  |  | BSI | 22 | 56.1 (2.46) | 51 | 54.7 (1.88) | 1.40 (0.25 - 2.55) |
| Follow-Up | F-PST | Wade et al (2019a)^4^ | CES-D | 22 | 11.8 (2.03) | 48 | 13.2 (1.58) | -1.40 (-2.36 to -0.44) |
|  |  |  | BSI | 22 | 51.7 (2.42) | 48 | 51.3 (1.92) | 0.40 (-0.75 to 1.55) |

a: Sum of n at trial level

Abbreviations: CES-D, Center for Epidemiological Studies Depression Scale; BSI, Brief Symptom Inventory

Comparison of Family Functioning Outcomes

A total of 12 measures across 1 publication reporting on 1 trial (PAAS trial^43^) were used for family functioning outcomes at post-test (see Multimedia Appendix 4 for measures used).

We calculated MDs and CIs for outcome measures for self-guided digital and face-to-face delivery modalities of 1 trial (PAAS^43^) (Multimedia Appendix 6). One outcome, parent-reported Discussion Quality, was significantly higher in the face-to-face versus self-guided digital delivery conditions at post-test, indicating higher discussion quality in the face-to-face delivery condition compared to the digital delivery condition. No other significant differences were found, all other CIs fell within and exceeded the minimal important difference.

**References**

1. Harris MA, Freeman KA, Duke DC. Seeing Is Believing: Using Skype to Improve Diabetes Outcomes in Youth. *Diabetes Care*. Aug 2015;38(8):1427-34. doi:10.2337/dc14-2469
2. Kurowski BG, Taylor HG, McNally KA, et al. Online Family Problem-Solving Therapy (F-PST) for Executive and Behavioral Dysfunction After Traumatic Brain Injury in Adolescents: A Randomized, Multicenter, Comparative Effectiveness Clinical Trial. *J Head Trauma Rehabil*. May/Jun 2020;35(3):165-174. doi:10.1097/htr.0000000000000545
3. Wade SL, Cassedy AE, Sklut M, et al. The Relationship of Adolescent and Parent Preferences for Treatment Modality With Satisfaction, Attrition, Adherence, and Efficacy: The Coping With Head Injury Through Problem-Solving (CHIPS) Study. *J Pediatr Psychol*. Apr 1 2019b;44(3):388-401. doi:10.1093/jpepsy/jsy087
4. Wade SL, Cassedy AE, McNally KA, et al. A Randomized Comparative Effectiveness Trial of Family-Problem-Solving Treatment for Adolescent Brain Injury: Parent Outcomes From the Coping with Head Injury through Problem Solving (CHIPS) Study. *J Head Trauma Rehabil*. Nov/Dec 2019a;34(6):E1-e9. doi:10.1097/htr.0000000000000487
5. Truttmann S, Philipp J, Zeiler M, et al. Long-Term Efficacy of the Workshop Vs. Online SUCCEAT (Supporting Carers of Children and Adolescents with Eating Disorders) Intervention for Parents: A Quasi-Randomised Feasibility Trial. *J Clin Med*. Jun 18 2020;9(6)doi:10.3390/jcm9061912
6. Murry VM, Kettrey HH, Berkel C, Inniss-Thompson MN. The Pathways for African American Success: Does Delivery Platform Matter in the Prevention of HIV Risk Vulnerability Among Youth? *J Adolesc Health*. Aug 2019b;65(2):255-261. doi:10.1016/j.jadohealth.2019.02.013
