# Supplementary material for: Comparing Digital Versus Face-to-Face Delivery of Systemic Psychotherapy Interventions: Systematic Review and Meta-Analysis of Randomized Controlled Trials
Source: Interact J Med Res. 2025 Feb 24;14:e46441. doi: 10.2196/46441 (PMC11894358; doi:10.2196/46441)
Supplement: Multimedia Appendix 10 [file ijmr_v14i1e46441_app10.docx]

**Multimedia Appendix 10:** Family functioning outcomes

**Table 1.** Means and mean differences of family functioning outcomes for face-to-face and self-guided delivery conditions at post-test (N=224^a^).

|  |  |  | Face-to-face delivery | | Digital delivery | | Mean difference (95% CI) |
| --- | --- | --- | --- | --- | --- | --- | --- |
| Trial | Publication | Outcome | n | Mean (SD) | n | Mean (SD) |  |
|  |  |  |  |  |  |  |  |
| PAAS | Murry et al., (2019b)^1^ | Y-OSFC | 72 | 33.51 (5.63) | 81 | 34.85 (4.85) | -1.34 (-3.02 to 0.34) |
|  |  | P-OSFC | 90 | 38.09 (5.40) | 110 | 37.48 (5.69) | 0.61 (-0.93 to 2.15) |
|  |  | Y-FoC | 100 | 7.65 (2.76) | 124 | 7.72 (2.65) | -0.07 (-0.78 to 0.64) |
|  |  | P-FoC | 99 | 10.64 (2.55) | 124 | 10.31 (2.53) | 0.33 (-0.34 to 1.00) |
|  |  | Y-DQ | 72 | 10.31 (3.23) | 81 | 11.01 (3.20) | -0.70 (-1.72 to 0.32) |
|  |  | P-DQ | 90 | 13.04 (2.99) | 110 | 12.29 (2.99) | 0.75 (-0.08 to 1.58) |
|  |  | Y-CiC | 72 | 1.75 (2.60) | 81 | 1.33 (2.04) | 0.42 (-0.33 to 1.17) |
|  |  | P-CiC | 90 | 1.99 (2.21) | 110 | 1.61 (2.30) | 0.38 (-0.25 to 1.01) |
|  |  | Y-AN/EaRE | 100 | 36.98 (7.31) | 124 | 36.19 (7.85) | 0.79 (-1.20 to 2.78) |
|  |  | P-AN/EaRE | 99 | 40.30 (7.09) | 124 | 41.58 (4.74) | -1.28 (-2.91 to 0.35) |

a: Sum of n at trial level

Abbreviations: Y-OSFC, Discussion Quality Scale, youth report; Y-FoC, Frequency of Conversation, youth report; Y-DQ, Parent-Youth Discussion Quality, youth report; Y-CiC, Conflicted an Ineffective Communication, youth report; P-OSFC, Discussion Quality Scale, parent report; P-FoC, Frequency of Conversation, parent report; P-DQ, Parent-Youth Discussion Quality, parent report; P-CiC, Conflicted an Ineffective Communication, parent report; Y-AN/EaRE, Articulated Norms and Expectations about Risk Engagement, youth report; P-AN/EaRE, Articulated Norms and Expectations about Risk Engagement, parent report

### **Reference**

1. Murry VM, Kettrey HH, Berkel C, Inniss-Thompson MN. The Pathways for African American Success: Does Delivery Platform Matter in the Prevention of HIV Risk Vulnerability Among Youth? *J Adolesc Health*. Aug 2019b;65(2):255-261. doi:10.1016/j.jadohealth.2019.02.013
