# Supplementary material for: Comparing Digital Versus Face-to-Face Delivery of Systemic Psychotherapy Interventions: Systematic Review and Meta-Analysis of Randomized Controlled Trials
Source: Interact J Med Res. 2025 Feb 24;14:e46441. doi: 10.2196/46441 (PMC11894358; doi:10.2196/46441)
Supplement: Multimedia Appendix 11 [file ijmr_v14i1e46441_app11.docx]

**Multimedia Appendix 11:** Adherence, attrition and further outcomes

**Table 1.** Comparison of further outcomes for face-to-face and therapist-guided digital delivery conditions at post-test and follow-up (N=537)

|  |  |  |  | Face-to-face delivery | | Digital delivery | | Mean difference (95% CI) |
| --- | --- | --- | --- | --- | --- | --- | --- | --- |
| Post-test/Follow-Up | Trial | Publication | Outcome | n | Mean (SD) | n | Mean (SD) |  |
|  |  |  |  |  |  |  |  |  |
| Post-test | BFST-D | Freeman et al., (2013)^1^ | Y-WAI | 39 | 210.78 (25.64^a^) | 32 | 215.88 (25.64)^a^ | -5.10 (-17.09 to 6.89^a^) |
|  |  |  | P-WAI | 39 | 221.62 (25.57^a^) | 32 | 224.84 (25.57^a^) | -3.22 (-15.17 to 8.73^a^) |
| Follow-up | F-PST | Wade et al (2019c)^2^ | P-PE | 22 | 9.0 (1.4) | 43 | 7.8 (2.1) | 1.20 (0.34- 2.06) |
|  |  |  | Y-PE | 23 | 8.2 (2.3) | 43 | 7.6 (2.1) | 0.60 (-0.53 to 1.73) |
|  |  |  | P-PB | 23 | 7.7 (1.2) | 43 | 7.5 (1.2) | 0.20 (-0.41 to 0.81) |
|  |  |  | Y-PB | 23 | 7.3 (2.1) | 43 | 7.5 (1.8) | -0.20 (-1.21 to 0.81) |
|  |  |  | P-OS | 23 | 8.6 (1.7) | 43 | 7.5 (2.2) | 1.10 (0.14- 2.06) |
|  |  |  | Y-OS | 23 | 7.2 (2.2) | 43 | 7.1 (2.4) | 0.10 (-1.05 to 1.25) |

^a^: Estimates of standard deviations based on provided *t*-statistic for between-group comparisons, the hand-calculated differences in means, and the n of participants for both groups. Estimate was calculated using the RevMan Calculator tool^3^.

Abbreviations: Y-WAI, Working Alliance Inventory (Client), youth report; P-WAI, Working Alliance Inventory (Client), parent report; P-PE, Programme Evaluation, parent report; Y-PE, Programme Evaluation, youth report; P-PB, Perceived Benefit, parent report; OS, Overall Satisfaction; P, Parent report; Y, Youth report.

**Table 2.** Comparison of further outcomes for face-to-face and self-guided digital delivery conditions at follow-up (N=519)

|  |  |  | Face-to-face delivery | | Digital delivery | | Mean difference (95% CI) |
| --- | --- | --- | --- | --- | --- | --- | --- |
| Trial | Study | Outcome | n | Mean (SD) | n | Mean (SD) |  |
|  |  |  |  |  |  |  |  |
| PAAS | Murry et al. (2018)^4^ | Number of sessions attended | 137 | 2 (2) | 138 | 4 (3) | -2 (-2.47 to -1.53) |
| F-PST | Kurowski et al (2020)^5^ | Number of sessions attended | 34 | 7 (4.5) | 60 | 7.1 (5.0) | -0.1 (-2.07 to 1.87) |
| F-PST | Wade et al (2019c)^2^ | P-PE | 22 | 9.0 (1.4) | 48 | 6.8 (2.2) | 2.20 (1.35-3.05) |
|  |  | Y-PE | 23 | 8.2 (2.3) | 48 | 7.0 (2.2) | 1.20 (0.07-2.33) |
|  |  | P-PB | 23 | 7.7 (1.2) | 48 | 7.3 (1.6) | 0.40 (-0.27 to 1.07) |
|  |  | Y-PB | 23 | 7.3 (2.1) | 48 | 7.7 (1.5) | -0.40 (-1.36 to 0.56) |
|  |  | P-OS | 23 | 8.6 (1.7) | 48 | 7.4 (2.1) | 1.20 (0.29-2.11) |
|  |  | Y OS | 23 | 7.2 (2.2) | 48 | 6.7 (2.4) | 0.50 (-0.63 to 1.63) |

Abbreviations: P-PE, Programme Evaluation, parent report; Y-PE, Programme Evaluation, youth report; P-PB, Perceived Benefit, parent report; OS, Overall Satisfaction; P, Parent report; Y, Youth report.

**Table 3.** Comparison of attrition for face-to-face and self-guided digital delivery conditions (N=369)

|  |  | Face-to-face delivery | | Digital delivery | | Risk Ratio (95% CI) |
| --- | --- | --- | --- | --- | --- | --- |
| Trial | Study | n | Number of drop-outs | n | Number of drop-outs |  |
|  |  |  |  |  |  |  |
| PAAS | Murry et al (2018)^4^ | 137 | 36 | 138 | 14 | 2.59 (1.46 - 4.58) |
| F-PST | Kurowski et al (2020)^5^ | 34 | 10 | 60 | 7 | 2.52 (1.06- 6.01) |

### References

1. Freeman KA, Duke DC, Harris MA. Behavioral health care for adolescents with poorly controlled diabetes via Skype: does working alliance remain intact? *J Diabetes Sci Technol*. May 1 2013;7(3):727-35. doi:10.1177/193229681300700318

2. Wade SL, Cassedy AE, Taylor HG, et al. Adolescent quality of life following family problem-solving treatment for brain injury. *J Consult Clin Psychol*. Nov 2019c;87(11):1043-1055. doi:10.1037/ccp0000440

3. Drahota A, Beller E. RevMan Calculator. Cochrane Training. 22/09/2022, Accessed 05/05/2022, <https://training.cochrane.org/resource/revman-calculator>

4. Murry VM, Berkel C, Liu N. The Closing Digital Divide: Delivery Modality and Family Attendance in the Pathways for African American Success (PAAS) Program. *Prev Sci*. Jul 2018;19(5):642-651. doi:10.1007/s11121-018-0863-z

5. Kurowski BG, Taylor HG, McNally KA, et al. Online Family Problem-Solving Therapy (F-PST) for Executive and Behavioral Dysfunction After Traumatic Brain Injury in Adolescents: A Randomized, Multicenter, Comparative Effectiveness Clinical Trial. *J Head Trauma Rehabil*. May/Jun 2020;35(3):165-174. doi:10.1097/htr.0000000000000545
